# The benefits and risks of adding PD-1/PD-L1 inhibitors to chemotherapy for stage IIIb-IV non-small-cell lung cancer: an updated meta-analysis based on phase 3 randomized controlled trials

**DOI:** 10.3389/fonc.2025.1590017

**Published:** 2025-09-11

**Authors:** Yun Xu, Baoliang Zhong, Chunlin Yu, Qingjian Hou, Wenying Chen, Wen Zheng, Wenxiong Zhang, Tonggang Zhou

**Affiliations:** ^1^ Department of Oncology, Shangrao People’s Hospital, Shangrao, China; ^2^ Department of Interventional Therapy, Ganzhou People’s Hospital, Ganzhou, China; ^3^ Department of Thoracic Surgery, The Second Affiliated Hospital, Jiangxi Medical College, Nanchang University, Nanchang, China

**Keywords:** PD-1/PD-L1 inhibitors, chemotherapy, non-small-cell lung cancer, meta-analysis, phase 3 randomized controlled trials

## Abstract

**Background:**

Previous research has confirmed that integrating PD-1/PD-L1 inhibitors with chemotherapy (PC) represents a more effective strategy for treating advanced non-small-cell lung cancer (NSCLC). However, with the increasing number of phase 3 randomized controlled trials (RCTs) published in recent years, it is essential to re-evaluate the validity of this conclusion and to comprehensively assess the efficacy and safety across diverse patient subgroups.

**Methods:**

We systematically reviewed phase 3 RCTs comparing PC with chemotherapy alone for stage IIIb-IV NSCLC. Data were extracted and analyzed for overall survival (OS), progression-free survival (PFS), response rates, and adverse events (AEs). Subgroup analyses were performed based on factors such as disease stage, pathological type, etc.

**Results:**

After screening, 19 phase 3 RCTs involving 9335 patients were included. Our updated analysis confirmed at PC therapy significantly improves OS (hazard ratio [HR]: 0.73 [0.69, 0.77], P < 0.00001), PFS (HR: 0.56 [0.52, 0.60], P < 0.00001), duration of response (DOR, HR: 0.50 [0.45, 0.54], P < 0.00001) and objective response rate (ORR, risk ratio [RR]: 1.59 [1.51, 1.67], P < 0.00001) compared to chemotherapy alone. The survival benefits were consistent across all subgroups and increases with longer follow-up. Brain metastases and PD-L1 combined positive score (CPS) > 50% were the favorable factors for PC group. However, the combined treatment was associated with an increased incidence of total/grade 3–5 treatment emergent AEs (TEAEs), and immune-related AEs (irAEs), although the overall safety profile remained manageable. The most common AEs in the PC group were blood toxicity related AEs (anemia, neutrophil count decreased, etc).

**Conclusion:**

The PC therapy continues to provide a substantial survival benefit for patients with stage IIIb-IV NSCLC. However, its higher incidence of AEs, especially irAEs, needs to be taken seriously.

**Systematic review registration:**

https://www.crd.york.ac.uk/PROSPERO/view/CRD420251005925, identifier CRD420251005925.

## Introduction

Non-small cell lung cancer (NSCLC) is a leading cause of cancer-related mortality globally, with advanced-stage disease often presenting limited treatment options and poor survival outcomes (1). One of the major challenges in current NSCLC treatment is the development of primary and acquired resistance to both chemotherapy and targeted therapies, which often leads to treatment failure. Moreover, conventional chemotherapy alone is limited by modest survival benefits and cumulative toxicity, while targeted therapies are only applicable to subsets of patients with specific driver mutations (2). The emergence of immune checkpoint inhibitors (ICIs), particularly those targeting the PD-1/PD-L1 pathway, has transformed the therapeutic landscape for advanced NSCLC. The rationale behind the PC regimen lies in its potential to overcome these limitations: chemotherapy not only reduces tumor burden but may also induce immunogenic cell death, thereby enhancing tumor antigen presentation, while PD-1/PD-L1 inhibitors restore T cell activity and help to counteract immune evasion and resistance mechanisms. By enhancing the immune system’s antitumor activity, these agents have demonstrated significant improvements in overall survival (OS) and progression-free survival (PFS) when administered in combination with chemotherapy (3).

However, the field is rapidly evolving, with numerous new phase 3 randomized controlled trials (RCTs) continually refining our understanding of these combinations (4–7). Recent studies have examined their efficacy across diverse patient subgroups, including those with varying PD-L1 expression levels, different histologic subtypes, and specific genetic mutations (4–7). While some trials reaffirm the superiority of PD-1/PD-L1 inhibitors combined with chemotherapy (PC), others report more nuanced outcomes, particularly in patients with low or negative PD-L1 expression, raising questions about universal applicability (8, 9). Meanwhile, safety remains a critical consideration. Immune-related adverse events (irAEs), such as pneumonitis and colitis, are well-documented risks of ICIs and may be exacerbated when combined with chemotherapy (10). Elderly patients and those with pre-existing autoimmune conditions are particularly susceptible, necessitating a careful evaluation of the risk-benefit profile in these populations (11).

Given the growing body of evidence, an updated meta-analysis is warranted to synthesize findings from recent phase 3 RCTs and provide a comprehensive evaluation of the benefits and risks of PC. This analysis aims to address key questions: (1) Does this combination continue to outperform chemotherapy alone in advanced NSCLC? (2) How do efficacy and safety profiles vary across patient subgroups? (3) What are the most frequent and severe adverse events (AEs) associated with these regimens? By integrating data from recent phase 3 RCTs, this meta-analysis seeks to offer evidence-based insights into optimizing treatment strategies for advanced NSCLC, ensuring that the benefits of these novel therapies are maximized while minimizing risks.

## Materials and methods

### Search strategy

Keywords including “PD-1/PD-L1 inhibitors”, “Lung cancer”, and “Randomized” were used in the search process. Six major databases-PubMed, ScienceDirect, Cochrane Library, Scopus, EMBASE, and Web of Science-were systematically searched. The investigation covered all available records from the inception of these databases up to February 13, 2025 (**Supplementary Table S1**).

### Selection criteria

The inclusion criteria: (1) Participants: patients diagnosed with stage IIIb-IV NSCLC; (2) Intervention and control: PC compared to chemotherapy; (3) Outcomes: survival, response rates, and AEs; (4) Study design: phase 3 RCTs.

We excluded studies if they were retrospective studies, letters, review articles, editorials, and conference abstracts.

### Data extraction

Two investigators independently collected data on study details (registration No., study name, etc), patient characteristics (age, pathological type, etc), survival outcomes (OS, PFS, etc), response rates (duration of response [DOR], objective response rate [ORR], etc), and AEs (treatment emergent AEs [TEAEs], irAEs, etc). In cases of missing data, corresponding authors were contacted for clarification, and discrepancies were resolved through re-evaluation by the investigators.

### Outcome assessments

OS and PFS were subgroup analyzed based on age, sex, race, ECOG PS, smoking status, pathological type, stage, brain metastases, liver metastases, PD-L1 combined positive score (CPS), PD-1/PD-L1 inhibitors type, and platinum chemotherapy type. If specific subgroup data were missing in individual studies, those studies were excluded from the relevant subgroup analysis but remained in the overall analyses. The survival rates of OS (OSR) and PFS (PFSR) were evaluated at 6 to 60 months, and the duration of response rate (DORR) were assessed at 6 to48 months.

### Quality assessment

Two instruments, the Cochrane Risk Assessment Tool and the Jadad scale, were used to assess the methodological quality of RCTs. The Jadad scale employs a 7-point scoring method, with scores ranging from 4 to 7 indicating high-quality studies (12, 13). Additionally, the outcomes were analyzed using the GRADE framework, which classifies evidence into four distinct levels: high, moderate, low, and very low (14).

### Statistical analysis

STATA 12.0 and Review Manager 5.3 were used to perform data analysis. For survival outcomes, hazard ratios (HR) were used, whereas risk ratios (RR) were utilized for dichotomous data. Different articles from the same trial were considered only if they reported distinct outcomes, while for the same outcome we used the most recent or most complete dataset. A fixed-effects model was used for low heterogeneity (I² < 50% or P > 0.1), whereas a random-effects model was applied when heterogeneity was higher. Meanwhile, for outcomes exhibiting substantial heterogeneity, sensitivity analyses were also conducted by sequentially excluding individual studies to evaluate the robustness of the pooled estimates. A P-value below 0.05 was considered statistically significant. Publication bias was examined using funnel diagrams, along with Egger’s and Begg’s statistical tests (15, 16).

## Results

### Search results

**Figure 1:**
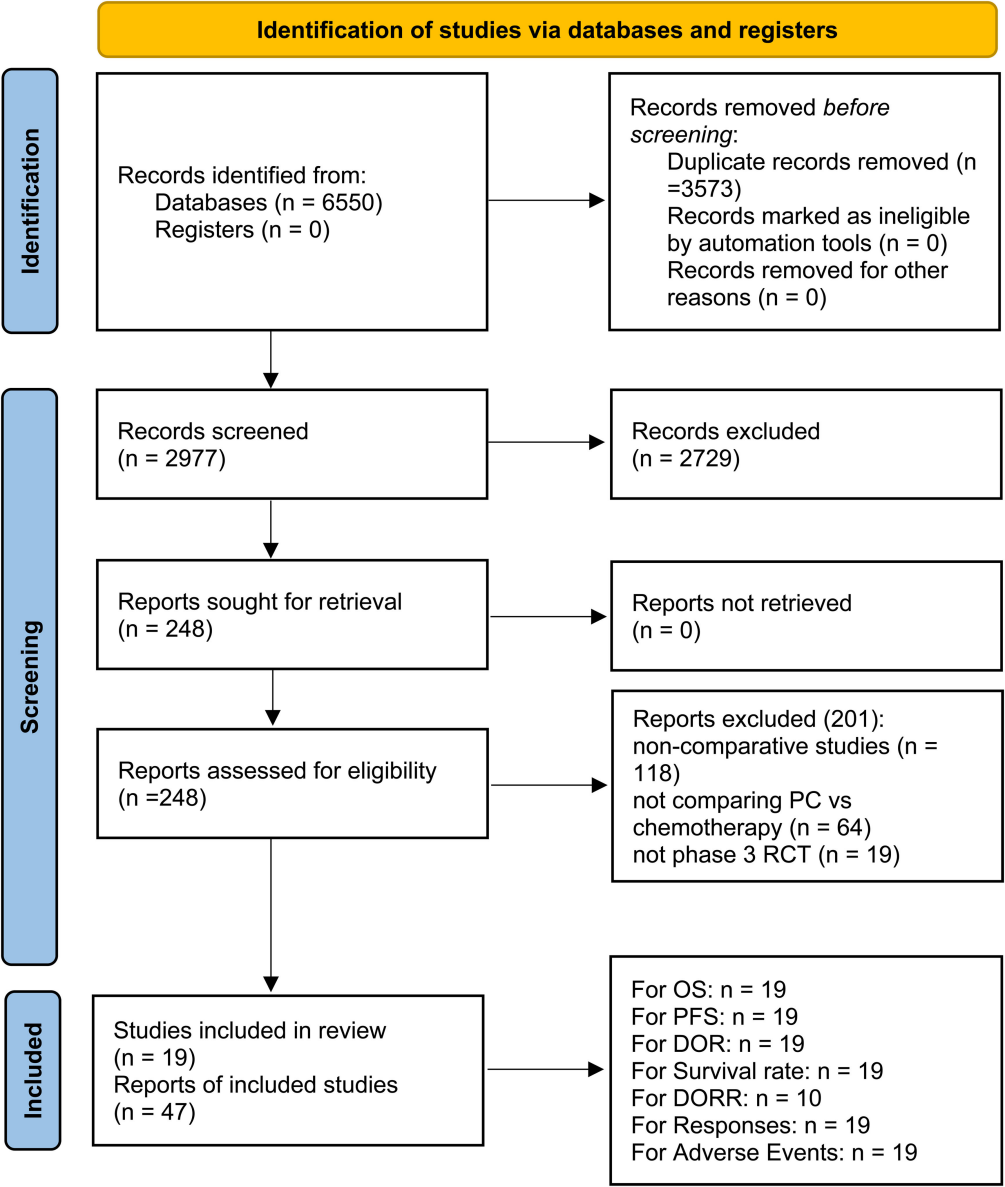
Flow chart.

Among the 2977 screened studies, 47 reports from 19 phase 3 RCTs (AK105-302, ASTRUM-004, CameL, CameL-Sq, CheckMate 227 Part 1b, CheckMate 227 Part 2, CHOICE-01, EMPOWER-Lung 3, GEMSTONE-302, IMpower130, IMpower131, IMpower132, KEYNOTE-189, KEYNOTE-407, ORIENT-11, ORIENT-12, POSEIDON, RATIONALE-304, and RATIONALE-307), encompassing a total of 9335 patients, were selected (**Figure 1**) (4–9, 17–57). **Table 1** summarizes the baseline characteristics of these studies. Of these, ten RCTs (5, 6, 8, 9, 21, 32–34, 37, 43) were international multicenter trials, while the remaining nine (4, 7, 17, 20, 30, 49, 52, 54, 56) were multicenter studies conducted in China. All included studies were considered high quality (**Supplementary Figure S1**, **Supplementary Table S2**). According to the GRADE framework, the evidence quality ranged from moderate to high (**Supplementary Table S3**).

**Table 1:** Baseline characteristics of the included studies.

Study	Registration no.	Period	Country	Groups	Patients	Sex(M/F)	Age (mean, year)	Pathological type		Stage		Mutation status	PD-1/PD-L1 type	Follow up (months)
								Sq	Non-sq	III	IV			
AK105–302 (4)	NCT03866993	2018.12-2020.10	China	PC	175	162/13	60.9	175	0	23	152	EGFR -/ALK -	Penpulimab	24.7
				Chemotherapy	175	162/13	61.9	175	0	26	149			
ASTRUM-004 (5)	NCT04033354	2019.08-2021.02	Global multicenter	PC	358	321/37	63	358	0	103	255	EGFR -/ALK -/ROS1-	Sugemalimab	31.1
				Chemotherapy	179	167/12	63	179	0	49	130			
CameL (17–19)	NCT03134872	2017.05-2018.06	China	PC	205	146/59	59	0	205	30	175	EGFR -/ALK -	Camrelizumab	65.2
				Chemotherapy	207	149/58	61	0	207	41	166			
CameL-Sq (20)	NCT03668496	2018.11-2019.12	China	PC	193	179/14	64	193	0	54	139	EGFR -/ALK -	Camrelizumab	13.5
				Chemotherapy	196	180/16	62	197	0	55	141			11.6
CheckMate 227 Part 1b (21–26)	NCT02477826	2015.08-2016.11	Global multicenter	PC	177	130/47	64	43	134	0	177	EGFR -/ALK -	Nivolumab	61.3
				Chemotherapy	186	125/61	64	46	140	0	186			
CheckMate 227 Part 2 (6)	NCT02477826	2017.01-2017.10	Global multicenter	PC	377	264/113	63	107	270	0	377	EGFR -/ALK -	Nivolumab	19.5
				Chemotherapy	378	266/112	64	105	273	0	378			
CHOICE-01 (7, 27)	NCT03856411	2019.04-2020.08	China	PC	309	247/62	63	147	162	49	260	EGFR -/ALK -	Toripalimab	21.2
				Chemotherapy	156	130/26	61	73	83	23	133			
EMPOWER-Lung 3 (8, 28, 29)	NCT03409614	2019.06-2020.09	Global multicenter	PC	312	268/44	63	133	179	45	267	EGFR -/ALK -/ROS1-	Cemiplimab	28.4
				Chemotherapy	154	123/31	63	67	87	24	130			
GEMSTONE-302 (30, 31)	NCT03789604	2018.12-2020.03	China	PC	320	254/66	62	129	191	0	320	EGFR -/ALK -/ROS1-/RET-	Sugemalimab	25.6
				Chemotherapy	159	129/30	64	63	96	0	159			
IMpower130 (32)	NCT02367781	2015.04-2017.02	Global multicenter	PC	451	266/185	64	0	451	0	451	EGFR -/ALK -	Atezolizumab	18.5
				Chemotherapy	228	134/94	65	0	228	0	228			19.2
IMpower131 (33)	NCT02367794	2015.06-2017.03	Global multicenter	PC	343	280/63	65	343	0	0	343	EGFR -/ALK -	Atezolizumab	18.1
				Chemotherapy	340	277/63	65	340	0	0	340			16.1
IMpower132 (34, 35)	NCT02657434	2016.04-2017.03	Global multicenter	PC	292	192/100	64	0	292	0	292	EGFR -/ALK -	Atezolizumab	14.8
				Chemotherapy	286	192/94	63	0	286	0	286			
KEYNOTE-189 (36–42)	NCT02578680	2016.02-2017.03	Global multicenter	PC	410	254/156	65	0	410	0	410	EGFR -/ALK -	Pembrolizumab	64.6
				Chemotherapy	206	109/97	64	0	206	0	206			
KEYNOTE-407 (43–48)	NCT02775435	2016.08-2017.12	Global multicenter	PC	278	220/58	65	278	0	0	278	EGFR -/ALK -	Pembrolizumab	56.9
				Chemotherapy	281	235/46	65	281	0	0	281			
ORIENT-11 (49–51)	NCT03607539	2018.08-2019.07	China	PC	266	204/62	61	0	266	21	245	EGFR -/ALK -	Sintilimab	30.8
				Chemotherapy	131	99/32	61	0	131	15	116			
ORIENT-12 (52)	NCT03629925	2018.08-2019.07	China	PC	179	163/16	64	179	0	39	140	EGFR -/ALK -	Sintilimab	8.0
				Chemotherapy	178	164/14	62	178	0	44	134			
POSEIDON (9, 53)	NCT03164616	2017.06-2018.09	Global multicenter	PC	338	253/85	65	128	210	0	338	EGFR -/ALK -	Durvalumab	63.4
				Chemotherapy	337	248/89	64	122	215	0	337			
RATIONALE 304 (54, 55)	NCT03663205	2018.07-2019.07	China	PC	223	168/55	60	0	223	40	183	EGFR -/ALK -	Tislelizumab	16.1
				Chemotherapy	111	79/32	61	0	111	24	87			
RATIONALE 307 (56, 57)	NCT03594747	2018.07-2019.06	China	PC	120	107/13	60	120	0	38	82	EGFR -/ALK -	Tislelizumab	20.5
				Chemotherapy	121	111/10	62	121	0	44	77			

ALK, Anaplastic lymphoma kinase; ECOG PS, Eastern Cooperative Oncology Group performance status; EGFR, Epidermal growth factor receptor; M/F, Male/Female; Non-sq, Non-squamous non-small-cell lung cancer; PD-1, Programmed death-1; PD-L1, Programmed death-ligand 1; PC, PD-1/PD-L1 inhibitors combined with chemotherapy; RET, Rearranged during transfection; ROS1, ROS Proto-Oncogene 1, receptor tyrosine kinase; Sq, Squamous cell carcinoma.

### Survival

**Figure 2:**
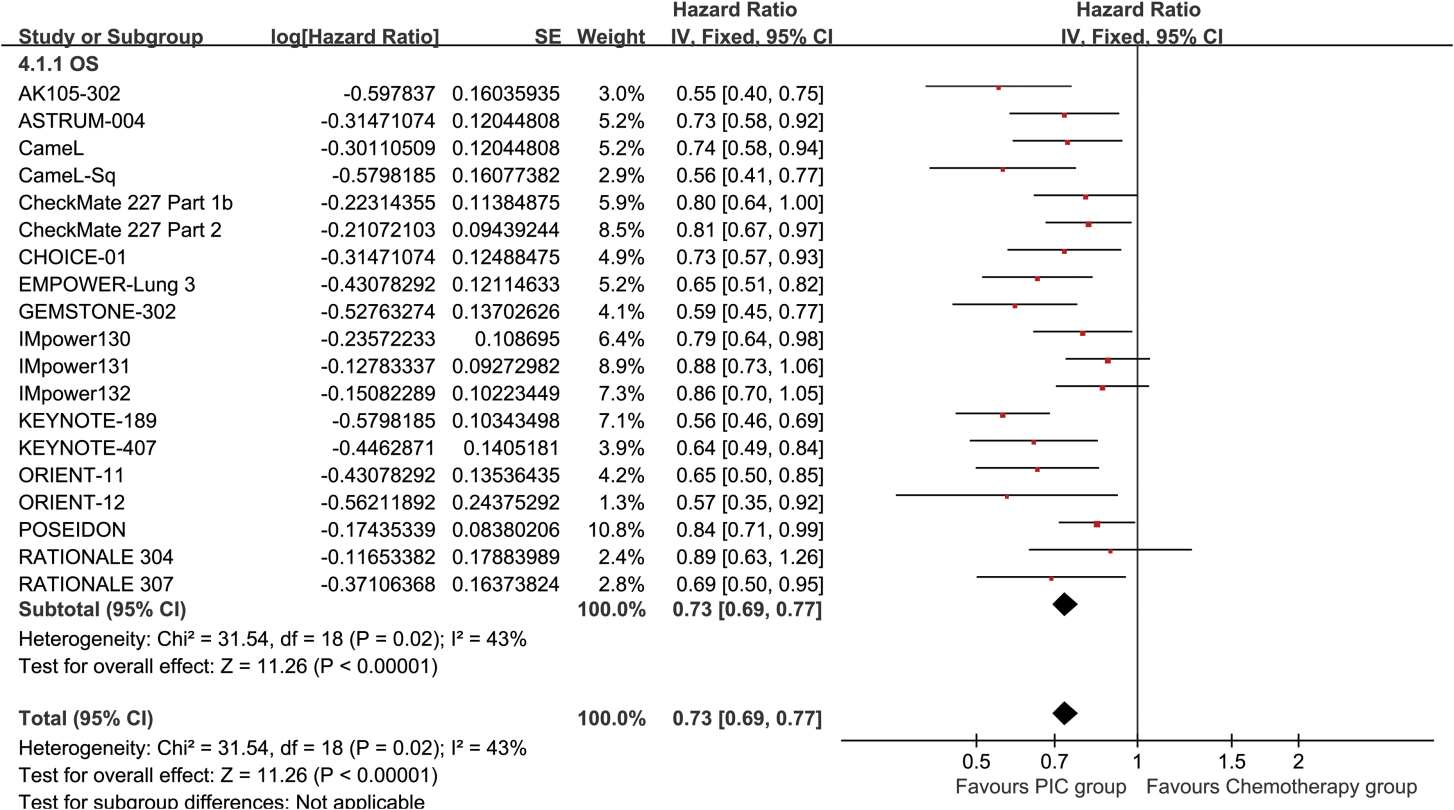
Forest plot of overall survival associated with PC versus chemotherapy.

**Figure 3:**
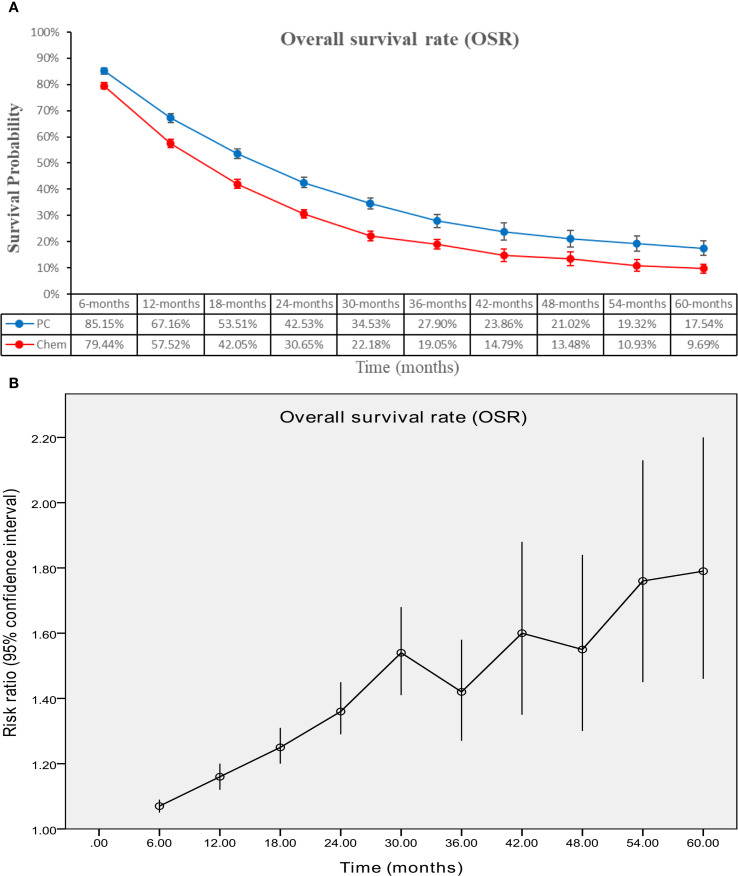
Comparisons of OSR associated with PC versus chemotherapy. **(A)** OSR at 6–60 months; **(B)** Trend of risk ratios in OSR.

The PC group demonstrated superior OS (HR: 0.73 [0.69, 0.77], P < 0.00001) (**Figure 2**). OSR showed a significant advantage for the PC group over a period of 6 to 60 months. The OS benefit became more pronounced as survival time extended (**Figure 3**; **Supplementary Figure S2**).

**Figure 4:**
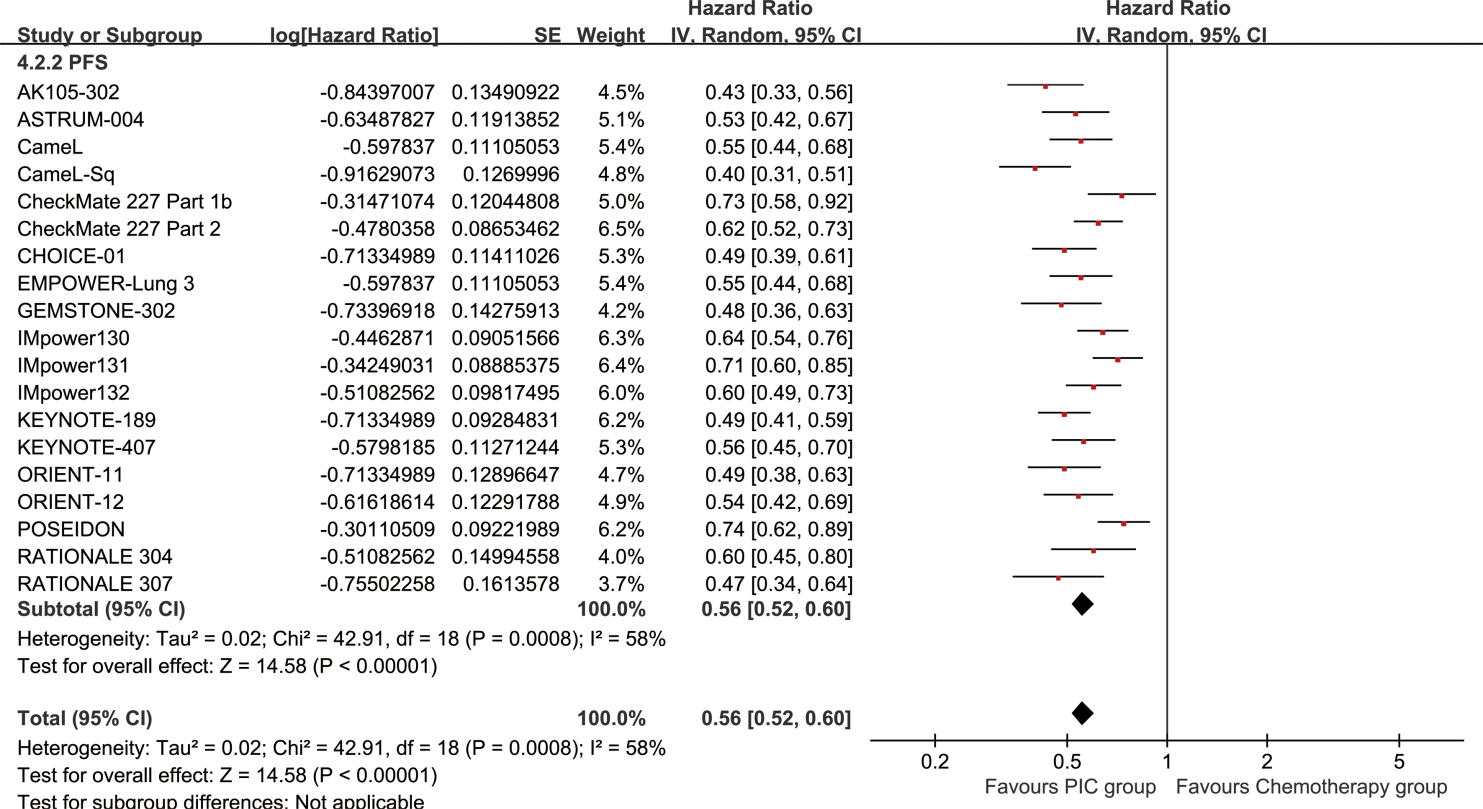
Forest plot of progression-free survival associated with PC versus chemotherapy.

**Figure 5:**
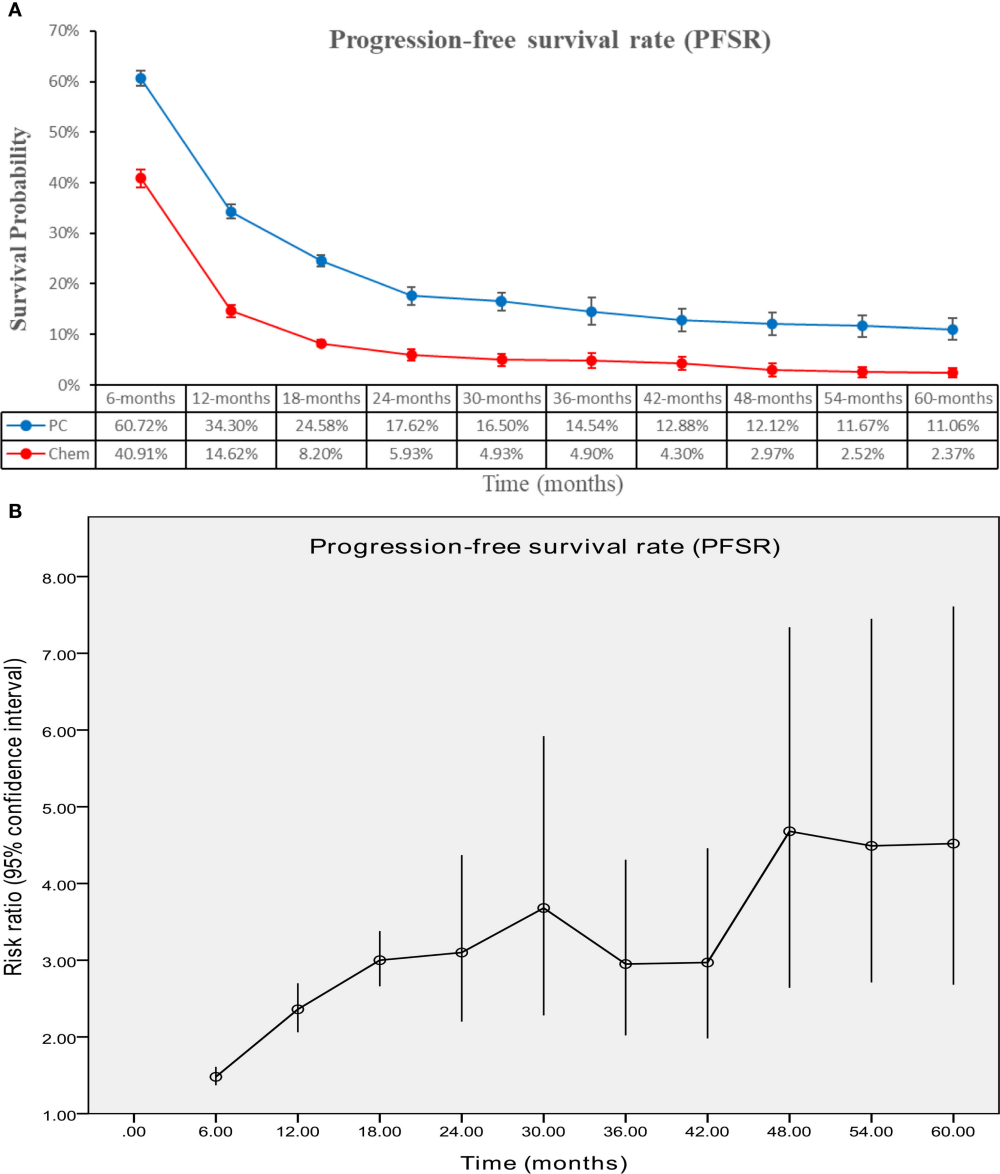
Comparisons of PFSR associated with PC versus chemotherapy. **(A)** PFSR at 6–60 months; **(B)** Trend of risk ratios in PFSR.

The PC group demonstrated enhanced PFS (HR: 0.56 [0.52, 0.60], P < 0.00001) (**Figure 4**). PFSR displayed a significant advantage for the PC group over a duration of 6 to 60 months. The PFS also benefit became more evident as survival time extended (**Figure 5**; **Supplementary Figure S3**).

### Subgroup analysis of survival

OS and PFS consistently favored PC in all subgroups (as described in the outcome assessments). Brain metastases and PD-L1 CPS > 50% were the favorable factors for PC group in both OS and PFS (**Table 2**).

**Table 2:** Subgroup analysis of overall survival and progression-free survival.

Subgroups	Overall survival				Progression-free survival			
	Included studies	Patients	HR (95% CI)	*P*	Included studies	Patients	HR (95% CI)	*P*
Total	19	9335	0.73 [0.69, 0.77]	< 0.00001	19	9335	0.56 [0.52, 0.60]	< 0.00001
Age								
< 65 years	14	4066	0.68 [0.59, 0.77]	< 0.00001	17	4568	0.52 [0.46, 0.59]	< 0.00001
> 65 years	14	3291	0.79 [0.72, 0.87]	< 0.00001	17	3648	0.59 [0.55, 0.64]	< 0.00001
Sex								
Female	13	1849	0.69 [0.61, 0.78]	< 0.00001	16	1819	0.58 [0.52, 0.65]	< 0.00001
Male	13	5159	0.76 [0.71, 0.82]	< 0.00001	16	6048	0.55 [0.49, 0.60]	< 0.00001
Race								
Asia	15	4136	0.69 [0.64, 0.76]	< 0.00001	14	4415	0.50 [0.47, 0.54]	< 0.00001
White	3	1380	0.78 [0.63, 0.97]	0.03	4	1558	0.66 [0.59, 0.73]	< 0.00001
ECOG PS								
0	14	2251	0.70 [0.63, 0.79]	< 0.00001	17	2300	0.53 [0.48, 0.59]	< 0.00001
1	14	5191	0.75 [0.70, 0.80]	< 0.00001	17	5902	0.56 [0.51, 0.61]	< 0.00001
Smoking status								
Current/former	12	4866	0.67 [0.60, 0.76]	< 0.00001	12	4984	0.51 [0.45, 0.58]	< 0.00001
Never	13	1125	0.82 [0.70, 0.96]	0.02	15	1282	0.62 [0.54, 0.71]	< 0.00001
Pathological type								
Squamous	13	4319	0.72 [0.67, 0.79]	< 0.00001	13	4279	0.53 [0.47, 0.60]	< 0.00001
Non-squamous	12	5054	0.73 [0.68, 0.79]	< 0.00001	12	5053	0.59 [0.55, 0.63]	< 0.00001
Stage								
III	6	396	0.70 [0.52, 0.96]	0.02	9	713	0.42 [0.35, 0.51]	< 0.00001
IV	15	7392	0.71 [0.65, 0.78]	< 0.00001	18	8210	0.57 [0.52, 0.61]	< 0.00001
Brain metastases								
Yes	6	363	0.61 [0.47, 0.79]	0.0002	6	319	0.41 [0.31, 0.55]	< 0.00001
No	7	3079	0.70 [0.65, 0.77]	< 0.00001	7	2707	0.52 [0.47, 0.57]	< 0.00001
Liver metastases								
Yes	7	673	0.84 [0.70, 1.00]	0.05	10	675	0.71 [0.60, 0.85]	0.0002
No	6	3012	0.78 [0.68, 0.91]	0.0009	9	3869	0.56 [0.52, 0.60]	< 0.00001
PD-L1 CPS								
<1%	14	3393	0.80 [0.73, 0.87]	< 0.00001	17	3706	0.69 [0.63, 0.75]	< 0.00001
>1%	9	2711	0.68 [0.61, 0.75]	< 0.00001	11	2976	0.48 [0.44, 0.52]	< 0.00001
1%-49%	10	2030	0.70 [0.62, 0.80]	< 0.00001	13	2282	0.55 [0.50, 0.62]	< 0.00001
>50%	11	1416	0.61 [0.52, 0.71]	< 0.00001	14	1757	0.45 [0.39, 0.50]	< 0.00001
PD-1/PD-L1 inhibitors type								
Penpulimab	1	350	0.55 [0.40, 0.75]	0.0002	1	350	0.43 [0.33, 0.56]	< 0.00001
Sugemalimab	2	1016	0.67 [0.56, 0.79]	< 0.00001	2	1016	0.50 [0.43, 0.59]	< 0.00001
Camrelizumab	2	801	0.67 [0.55, 0.81]	< 0.0001	2	801	0.47 [0.35, 0.64]	< 0.00001
Nivolumab	2	1118	0.81 [0.70, 0.93]	0.003	2	1118	0.66 [0.57, 0.75]	< 0.00001
Toripalimab	1	465	0.73 [0.57, 0.93]	0.01	1	465	0.49 [0.39, 0.61]	< 0.00001
Cemiplimab	1	466	0.65 [0.51, 0.82]	0.0004	1	466	0.55 [0.44, 0.68]	< 0.00001
Atezolizumab	3	1940	0.85 [0.76, 0.95]	0.004	3	1940	0.65 [0.59, 0.72]	< 0.00001
Pembrolizumab	2	1175	0.59 [0.50, 0.69]	< 0.00001	2	1175	0.52 [0.45, 0.60]	< 0.00001
Sintilimab	2	794	0.63 [0.50, 0.79]	< 0.0001	2	754	0.52 [0.43, 0.61]	< 0.00001
Durvalumab	1	675	0.84 [0.71, 0.99]	0.04	1	675	0.74 [0.62, 0.89]	0.001
Tislelizumab	2	575	0.77 [0.61, 0.98]	0.03	2	575	0.54 [0.43, 0.66]	< 0.00001
Platinum chemotherapy type								
Cisplatin	3	501	0.65 [0.44, 0.95]	0.02	4	636	0.55 [0.46, 0.66]	< 0.00001
Carboplatin	14	5851	0.72 [0.67, 0.77]	< 0.00001	15	6073	0.54 [0.50, 0.57]	< 0.00001

CI, Confidence interval; CPS, Combined positive score; ECOG PS, Eastern Cooperative Oncology Group Performance Status; HR, Hazard ratio; OS, Overall survival; PC, PD-1/PD-L1 inhibitors combined with chemotherapy; PD, Progressive disease; PD-1, Programmed cell death protein 1; PD-L1, Programmed death-ligand 1; PFS, Progression-free survival.

### Responses

**Figure 6:**
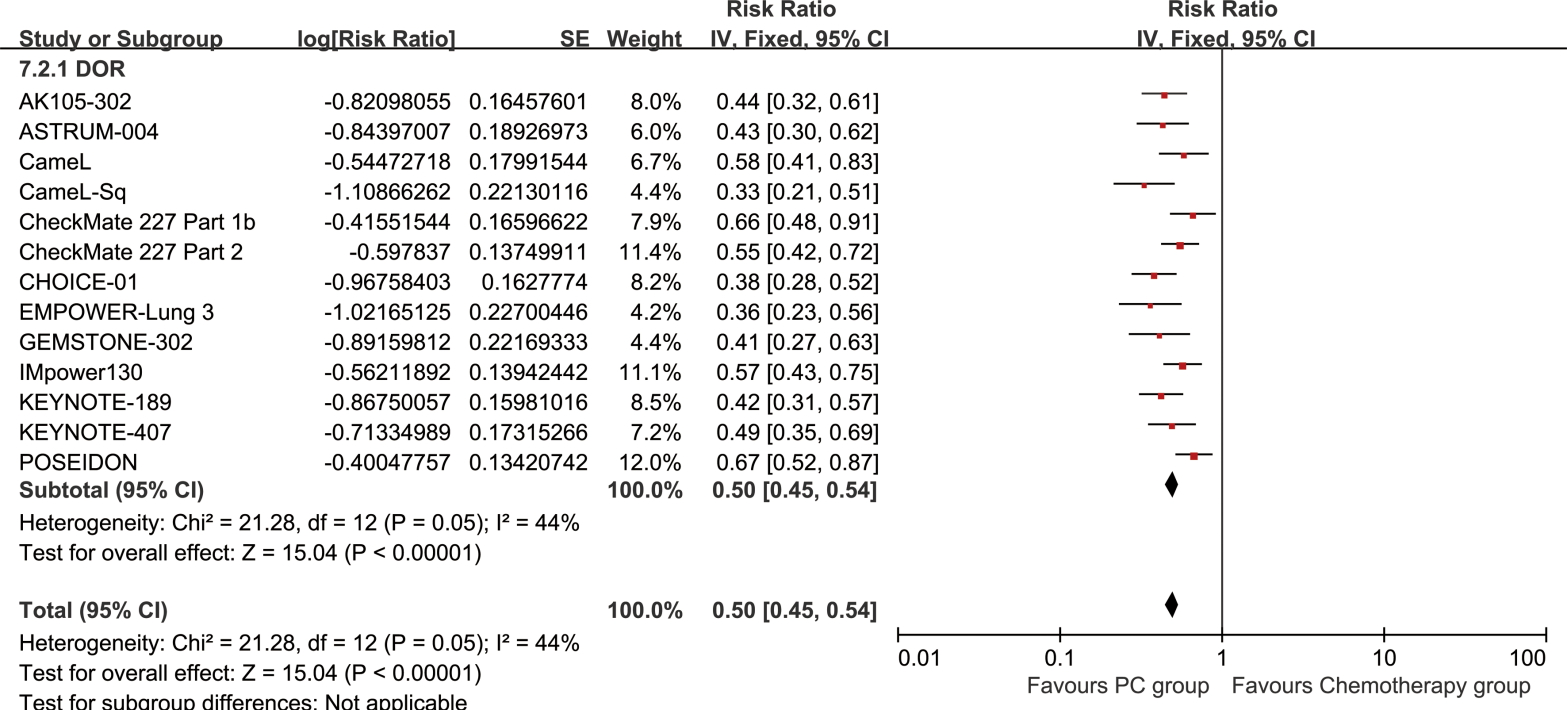
Forest plot of duration of response associated with PC versus chemotherapy.

**Figure 7:**
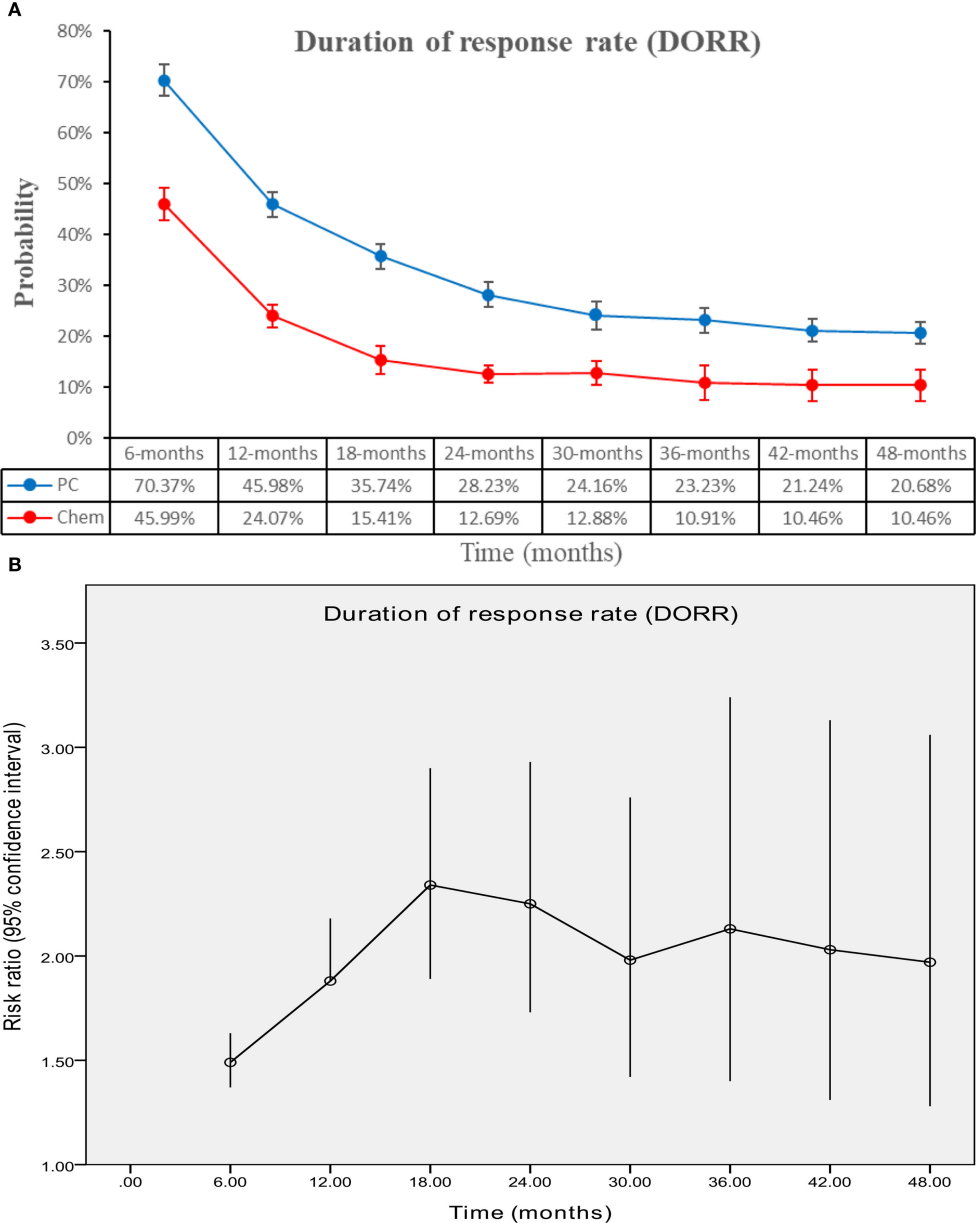
Comparisons of DORR associated with PC versus chemotherapy. **(A)** DORR at 6–48 months; **(B)** Trend of risk ratios in DORR.

The PC group exhibited superior DOR (HR: 0.50 [0.45, 0.54], P < 0.00001) (**Figure 6**). DORR consistently favored the PC group over a period of 6 to 48 months (**Figure 7**; **Supplementary Figure S4**).

The PC group achieved superior ORR (RR: 1.59 [1.51, 1.67], P < 0.00001), disease control rate (DCR) (RR: 1.12 [1.07, 1.18], P < 0.00001), complete response (CR) (RR: 2.30 [1.64, 3.23], P < 0.00001), and partial response (PR) (RR: 1.55 [1.47, 1.64], P < 0.00001). In contrast, the chemotherapy group had higher rates of stable disease (SD) (RR: 1.59 [1.51, 1.67], P < 0.00001) and progressive disease (PD) (RR: 1.55 [1.47, 1.64], P < 0.00001) (**Table 3**; **Supplementary Figure S5**).

**Table 3:** Tumor Responses.

Responses	PC		Chemotherapy		Risk ratio [95% CI]	P
	Event/total	%	Event/total	%		
ORR	2851/5326	53.53%	1348/4009	33.62%	1.59 [1.51, 1.67]	< 0.00001
DCR	3737/4376	85.40%	2433/3227	75.40%	1.12 [1.07, 1.18]	< 0.00001
CR	127/4668	2.72%	45/3513	1.28%	2.30 [1.64, 3.23]	< 0.00001
PR	2384/4668	51.07%	1160/3513	33.02%	1.55 [1.47, 1.64]	< 0.00001
SD	1354/4376	30.94%	1382/3227	42.83%	0.71 [0.65, 0.78]	< 0.00001
PD	344/4376	7.86%	454/3227	14.07%	0.55 [0.48, 0.63]	< 0.00001

CI, Confidence interval; CR, Complete response; DCR, Disease control rate; ORR, Objective response rate; PC, PD-1/PD-L1 inhibitors combined with chemotherapy; PD, Progressive disease; PD-1, Programmed cell death protein 1; PD-L1, Programmed death-ligand 1; PR, Partial response; RR, Risk ratio; SD, Stable disease.

### Safety

Overall, the PC group experienced higher incidences of total TEAEs/TRAEs/irAEs, grade 3–5 TEAEs/TRAEs/irAEs, serious TEAEs/TRAEs/irAEs, TEAEs/TRAEs/irAEs leading to discontinuation, and TRAEs/irAEs leading to death (**Table 4**).

**Table 4:** Summary of adverse events.

Adverse events	PC		Chemotherapy		Risk ratio [95% CI]	P
	Event/total	%	Event/total	%		
TEAEs						
Total TEAEs	5178/5326	97.22%	3793/4009	94.61%	1.01 [1.00, 1.02]	0.03
Grade 3–5 TEAEs	3606/5326	67.71%	2404/4009	59.97%	1.10 [1.05, 1.15]	< 0.0001
Serious TEAEs	1481/3504	42.27%	774/2681	28.87%	1.43 [1.26, 1.62]	< 0.00001
TEAEs leading to discontinuation	1081/5326	20.30%	479/4009	11.95%	1.65 [1.40, 1.94]	< 0.00001
TEAEs leading to death	375/4949	7.58%	235/3631	6.47%	1.20 [1.02, 1.40]	0.02
TRAEs						
Total TRAEs	3969/4349	91.26%	3025/3433	88.12%	1.03 [1.01, 1.06]	0.007
Grade 3–5 TRAEs	2384/4349	54.82%	1660/3433	48.35%	1.14 [1.06, 1.23]	0.0003
Serious TRAEs	725/2852	25.42%	375/2293	16.35%	1.55 [1.27, 1.90]	< 0.0001
TRAEs leading to discontinuation	450/3143	14.32%	162/2458	6.59%	2.17 [1.83, 2.58]	< 0.00001
TRAEs leading to death	100/4229	2.36%	52/3312	1.57%	1.58 [1.13, 2.21]	0.007
irAEs						
Total irAEs	1312/3719	35.28%	404/2548	15.86%	2.59 [1.94, 3.47]	< 0.00001
Grade 3–5 irAEs	341/3719	9.17%	77/2548	3.02%	3.04 [2.38, 3.87]	< 0.00001
Serious irAEs	31/513	6.04%	6/512	1.17%	5.16 [2.17, 12.25]	0.0002
irAEs leading to discontinuation	20/513	3.90%	3/512	0.59%	6.65 [1.99, 22.25]	0.002
irAEs leading to death	11/1957	0.56%	1/1545	0.06%	3.19 [0.89, 11.41]	0.07

AE, Adverse event; CI, Confidence interval; irAE, Immune-related adverse event; PC, PD-1/PD-L1 inhibitors combined with chemotherapy; PD-1, Programmed cell death protein 1; PD-L1, Programmed death-ligand 1; RR, Risk ratio; TEAE, Treatment-emergent adverse event; TRAE, Treatment-related adverse event.

In TEAE analysis, the PC group showed higher occurrence of any grade nausea, alopecia, platelet count decreased, fatigue, alanine ALT increased, AST increased, decreased appetite, constipation, diarrhea, vomiting, pyrexia, hypoalbuminaemia, rash, arthralgia, edema peripheral, peripheral sensory neuropathy, pruritus, hyperglycemia, hypothyroidism, pneumonia, blood creatinine increased, hyperthyroidism, hypercholesteraemia, and interstitial lung disease (**Supplementary Table S4**). Meanwhile, the PC group also experienced higher rates of grade 3–5 platelet count decreased, fatigue, decreased appetite, diarrhea, arthralgia, and rash (**Supplementary Table S5**).

In irAEs analysis, the PC group showed higher occurrence of any grade hypothyroidism, pneumonitis, pneumonia, hepatitis, hyperthyroidism, severe skin reactions, infusion reactions, colitis, nephritis, adrenal insufficiency, and pancreatitis (**Supplementary Table S6**). Meanwhile, the PC group also experienced higher rates of grade 3–5 hepatitis, pneumonitis, severe skin reactions, colitis, hypothyroidism, and nephritis (**Supplementary Table S7**).

### Sensitivity analysis

The findings for PFS, DCR, and total TEAEs remained robust after excluding individual studies in the sensitivity analysis (**Supplementary Figure S6**).

### Publication bias

Funnel plots for OS, PFS, ORR, and grade 3–5 TEAEs appeared symmetrical, suggesting an acceptable level of publication bias (**Supplementary Figure S7**). Furthermore, no significant publication bias was found by Egger’s and Begg’s tests for these outcomes (all p > 0.05) (**Supplementary Figure S8**).

## Discussion

The PC therapy has revolutionized the treatment landscape for advanced NSCLC, particularly for stage IIIb-IV disease. However, the rapid evolution of immunotherapy and the publication of numerous phase 3 RCTs in recent years have introduced new complexities and controversies (4–7). Given the persistent challenges of drug resistance and the limitations of current therapies, the PC regimen offers a rational approach by combining cytotoxic and immune-mediated mechanisms to achieve more durable responses. While earlier studies established the superiority of PC over chemotherapy alone, emerging evidence indicates that the benefits may vary across patient subgroups, particularly those with low or negative PD-L1 expression (17, 20). Additionally, the safety profile of PC, especially the incidence of irAEs, remains a critical concern, particularly for vulnerable populations such as elderly patients or those with pre-existing autoimmune conditions (21, 27). These uncertainties underscore the need for an updated meta-analysis to synthesize the latest evidence and conduct a thorough assessment of PC’s effectiveness and safety in advanced NSCLC. This updated meta-analysis, encompassing 19 phase 3 RCTs and 9,335 patients, confirms the significant survival benefits of PC over chemotherapy alone. The pooled results demonstrate substantial improvements in OS, PFS, DOR, and ORR. Subgroup analyses further reveal that patients with brain metastases and those with a PD-L1 CPS > 50% derive the greatest benefit from PC. Nevertheless, the combined treatment showed increased occurrences of AEs, including TEAEs, TRAEs, and irAEs, necessitating careful risk-benefit assessment in clinical practice.

The survival benefits of PC in advanced NSCLC are robust, as evidenced by marked improvements in OS (HR: 0.73) and PFS (HR: 0.56) in our meta-analysis. These findings align with recent studies, such as the KEYNOTE-189 and IMpower130 trials, which reported similar hazard ratios for OS and PFS in favor of PC (35, 37). Notably, the survival benefits of PC appear to increase over time, with OS and PFS rates showing greater divergence between the PC and chemotherapy groups at longer follow-up intervals. This suggests that the immunomodulatory effects of PD-1/PD-L1 inhibitors may provide durable clinical benefits, a phenomenon also observed in other malignancies treated with immune checkpoint inhibitors (4, 20). Subgroup analyses further illuminate the differential efficacy of PC across patient populations. Patients with brain metastases, a historically poor prognostic group, exhibited particularly pronounced survival benefits from PC. This finding is consistent with recent studies highlighting the potential of immunotherapy to penetrate the blood-brain barrier and exert antitumor effects in the central nervous system (37, 49). Meanwhile, all evaluated PD-1/PD-L1 inhibitors confer OS and PFS benefits versus chemotherapy, supporting a class effect in advanced NSCLC. While numerical differences in pooled HRs are apparent across agents, these arise from indirect, across-trial contrasts with heterogeneous populations, backbones, PD-L1 assays, and follow-up durations. Notably, patients with PD-L1 CPS >50% have consistently demonstrated a greater magnitude of benefit from immunotherapy-based regimens, highlighting the potential of CPS as a predictive biomarker in clinical decision-making. However, variability in testing methods and thresholds remains a challenge for universal application (58). In our subgroup analysis, patients with elevated PD-L1 expression (CPS > 50%) showed greater survival advantages, underscoring PD-L1 as a key predictor of immunotherapy effectiveness. In contrast, the survival benefits in patients with low or negative PD-L1 expression, though statistically significant, were less pronounced, raising considerations regarding the cost-effectiveness of PC in this subgroup (37, 39). Tumor mutational burden (TMB) has emerged as another promising biomarker, as tumors with high TMB tend to harbor more neoantigens, which can enhance immune recognition and response to immune checkpoint inhibitors. Recent evidence suggests that TMB may serve as an independent predictor of treatment efficacy across multiple cancer types. Incorporating both PD-L1 CPS and TMB into predictive models may improve the precision of patient stratification in future clinical trials (59). These findings highlight the need for further research to identify additional biomarkers that can refine patient selection for PC. Enhanced DOR further reinforces PC’s survival advantage, with a significantly prolonged duration in the PC group (HR: 0.50). This suggests that PC not only delays disease progression but also sustains tumor control over a more extended period, a key factor in improving long-term outcomes. The durability of response is particularly important in the context of immunotherapy, where the immune system’s memory effect can lead to prolonged antitumor activity even after treatment discontinuation (17, 21). Although our meta-analysis focuses on clinical outcomes, emerging preclinical and translational studies provide insight into the potential mechanisms underlying the superior efficacy of PD-1/PD-L1 inhibitors combined with chemotherapy. Chemotherapy can enhance tumor immunogenicity by increasing neoantigen presentation and promoting immunogenic cell death, thereby synergizing with PD-1/PD-L1 blockade to enhance cytotoxic T-cell responses. Furthermore, PC therapy has been shown to modulate the tumor microenvironment by reducing immunosuppressive cells such as regulatory T cells and myeloid-derived suppressor cells, and by increasing the infiltration and activation of effector CD8+ T cells. Cytokine profiling studies have also suggested that combined therapy may augment pro-inflammatory cytokine production, contributing to durable antitumor responses (60). Recent studies have further broadened the landscape of NSCLC research in ways that may intersect with immunotherapy. For example, analysis of bronchoalveolar lavage fluid microbiota has revealed significant associations with prognosis and immune modulation in NSCLC, while novel agents such as cycloastragenol have shown antitumor efficacy through apoptosis and autophagy pathways, potentially enhancing immunotherapeutic responses. These findings highlight the need to integrate clinical, microbiological, and molecular perspectives to optimize future immunotherapy-based strategies (61, 62).

In addition to survival outcomes, this meta-analysis highlights the superior response rates associated with PC. The PC group exhibited an ORR approximately 60% higher than the chemotherapy group (RR: 1.59), with similar improvements observed in DCR, CR, and PR rates. These findings are consistent with recent trials, such as the ORIENT-11 and RATIONALE-307 studies, which reported ORRs exceeding 60% in the PC arms (49, 56). The improved response rates likely contribute to the observed survival benefits, as deeper and more durable responses are associated with prolonged disease control and delayed progression. The DOR, a key measure of treatment response durability, was notably extended in the PC group (HR: 0.50). This finding aligns with the hypothesis that immunotherapy enhances the immune system’s ability to maintain long-term tumor control, even after the cessation of treatment (9, 43). However, the higher rates of PD in the chemotherapy group suggest that PC may be particularly effective in preventing disease progression, a key determinant of survival in advanced NSCLC. The improved response rates and DOR observed in the PC group may also have implications for patient quality of life. Patients who achieve a complete or partial response are more likely to experience symptom relief and improved functional status, which are critical considerations in the management of advanced NSCLC (4, 17). Furthermore, the higher rates of disease control in the PC group may reduce the need for subsequent lines of therapy, thereby minimizing the cumulative toxicity associated with multiple treatment regimens.

While the efficacy of PC is well-established, its safety profile remains a critical consideration. Our meta-analysis verifies that PC leads to increased incidences of TEAEs, TRAEs, and irAEs relative to chemotherapy alone. The most common AEs in the PC group were hematologic toxicities, such as anemia and decreased neutrophil count, which are likely attributable to the chemotherapy component of the regimen. However, the higher incidence of irAEs, including pneumonitis, hepatitis, and colitis, underscores the unique toxicity profile of immunotherapy (54, 56). The higher occurrence of grade 3–5 AEs, especially irAEs, in the PC group underscores the importance of close monitoring and proactive toxicity management. Recent studies have emphasized the importance of multidisciplinary care teams and standardized protocols for managing irAEs, which can significantly reduce morbidity and mortality associated with these events (63). Additionally, the higher rates of treatment discontinuation and death due to TRAEs/irAEs in the PC group underscore the importance of patient selection and risk stratification, particularly for vulnerable populations such as elderly patients or those with pre-existing autoimmune conditions (49, 52). The safety profile of PC also has implications for treatment sequencing and combination strategies. For example, a greater occurrence of irAEs in the PC group might hinder the viability of future immunotherapy for patients with severe toxic effects. Although rare, severe irAEs such as myocarditis and Guillain–Barré syndrome were observed in the PC group. These findings underscore the importance of vigilant monitoring and long-term follow-up, as some rare toxicities may emerge late or be underreported in clinical trials (64). This underscores the need for personalized treatment approaches that balance the potential benefits of PC with the risks of toxicity, particularly in patients with comorbidities or poor performance status (58).

Compared with the study by Meng et al. (2022), which analyzed a broader NSCLC population and demonstrated consistent OS and PFS benefits across PD-L1 subgroups, our findings align in confirming the robust efficacy of ICI plus chemotherapy but also expand upon their work by including additional trials and updated evidence (65). In contrast, Chen et al. (2022) focused specifically on squamous NSCLC, reporting stronger relative benefits in OS and PFS, likely reflecting histology-specific sensitivity to chemo-immunotherapy, but with a higher incidence of hematologic and hepatic toxicities (66). These differences can be largely attributed to variations in patient populations (all NSCLC vs. squamous only), the scope of included trials, and the weighting of safety endpoints (67). Taken together, our meta-analysis complements prior work by providing a more comprehensive overview across different PD-1/PD-L1 inhibitors and NSCLC subtypes, while also highlighting that efficacy and safety profiles may vary depending on histology and study design.

Despite its comprehensive scope, this meta-analysis has several limitations. First, heterogeneity among the included studies in patient populations, treatment regimens, and follow-up durations might have impacted the pooled outcomes. Second, the subgroup analyses, while informative, were limited by the availability of data in the original studies. For example, the impact of specific genetic mutations, such as EGFR or ALK alterations, on the efficacy of PC could not be fully explored due to insufficient data. Third, the long-term safety profile of PC remains incompletely characterized, as many of the included studies had relatively short follow-up periods. Fourth, the meta-analysis was unable to assess the cost-effectiveness of PC, a important consideration for healthcare systems worldwide. Fifth, our study is limited by the lack of data on the optimal sequencing of PD-1/PD-L1 inhibitors and chemotherapy, as most trials used induction chemoimmunotherapy followed by maintenance immunotherapy. Sixth, a further limitation is the absence of direct RCTs comparing different PD-1/PD-L1 inhibitors; our agent-level subgroup results versus chemotherapy cannot be interpreted as between-agent comparisons. Seventh, another limitation is the potential bias in AE reporting across trials, as differences in grading systems, monitoring intensity, and reporting standards may affect the comparability of safety outcomes. Finally, the long-term incidence of rare irAEs could not be fully characterized due to limited follow-up in the included trials.

## Conclusion

This updated meta-analysis confirms the significant survival and response benefits of PC therapy in advanced NSCLC. The survival benefits were consistent across all subgroups (particularly effective in patients with brain metastases and high PD-L1 expression) and increases with the prolongation of survival time. Nevertheless, the increased occurrence of AEs, especially irAEs, requires careful patient selection and proactive toxicity control. Future studies should aim to discover novel biomarkers for better patient stratification and develop strategies to mitigate PC-related risks.

## Data Availability

The original contributions presented in the study are included in the article/**Supplementary Material**. Further inquiries can be directed to the corresponding author.

## Supplementary material

Supplementary Figure 1 Cochrane Risk Assessment.

Supplementary Figure 2 Forest plots of OSR at 6–60 months associated with PC versus chemotherapy.

Supplementary Figure 3 Forest plots of PFSR at 6–60 months associated with PC versus chemotherapy.

Supplementary Figure 4 Forest plots of DORR at 6–48 months associated with PC versus chemotherapy.

Supplementary Figure 5 Forest plots of responses associated with PC versus chemotherapy.

Supplementary Figure 6 Sensitivity analysis of PFS **(A)**, DCR **(B)**, and total TEAEs **(C)**.

Supplementary Figure 7 Funnel plots of OS **(A)**, PFS **(B)**, ORR **(C)**, and grade 3–5 TEAEs **(D)**.

Supplementary Figure 8 Egger’s and Begg’s tests of OS **(A)**, PFS **(B)**, and grade TEAEs **(C)**.
