# Supplementary figures and images for: The benefits and risks of adding PD-1/PD-L1 inhibitors to chemotherapy for stage IIIb-IV non-small-cell lung cancer: an updated meta-analysis based on phase 3 randomized controlled trials
Source: Front Oncol. 2025 Sep 11;15:1590017. doi: 10.3389/fonc.2025.1590017 (PMC12460147; doi:10.3389/fonc.2025.1590017)

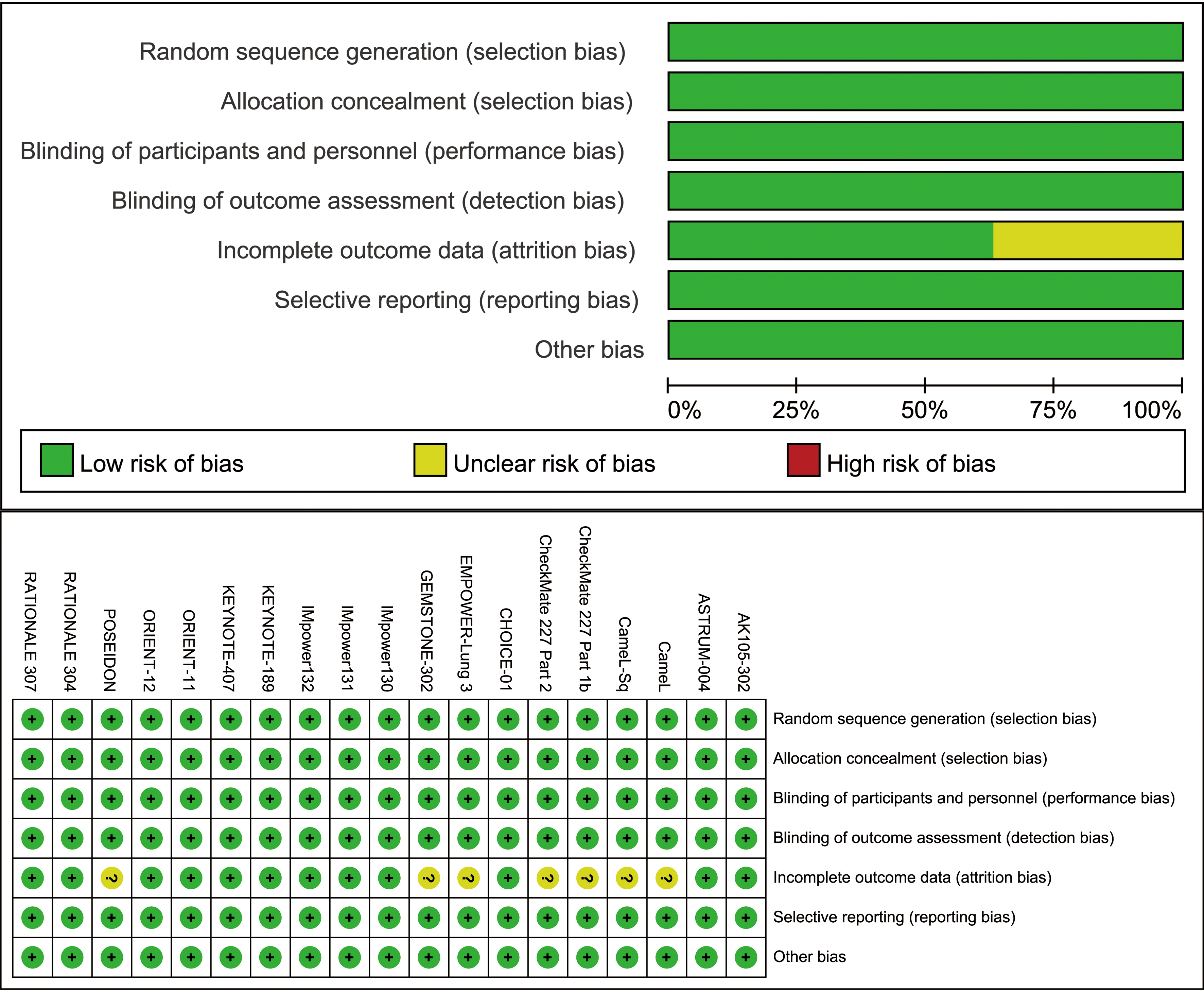

Supplement: Supplementary Figure 1 — Cochrane Risk Assessment. [file Image1.tif]

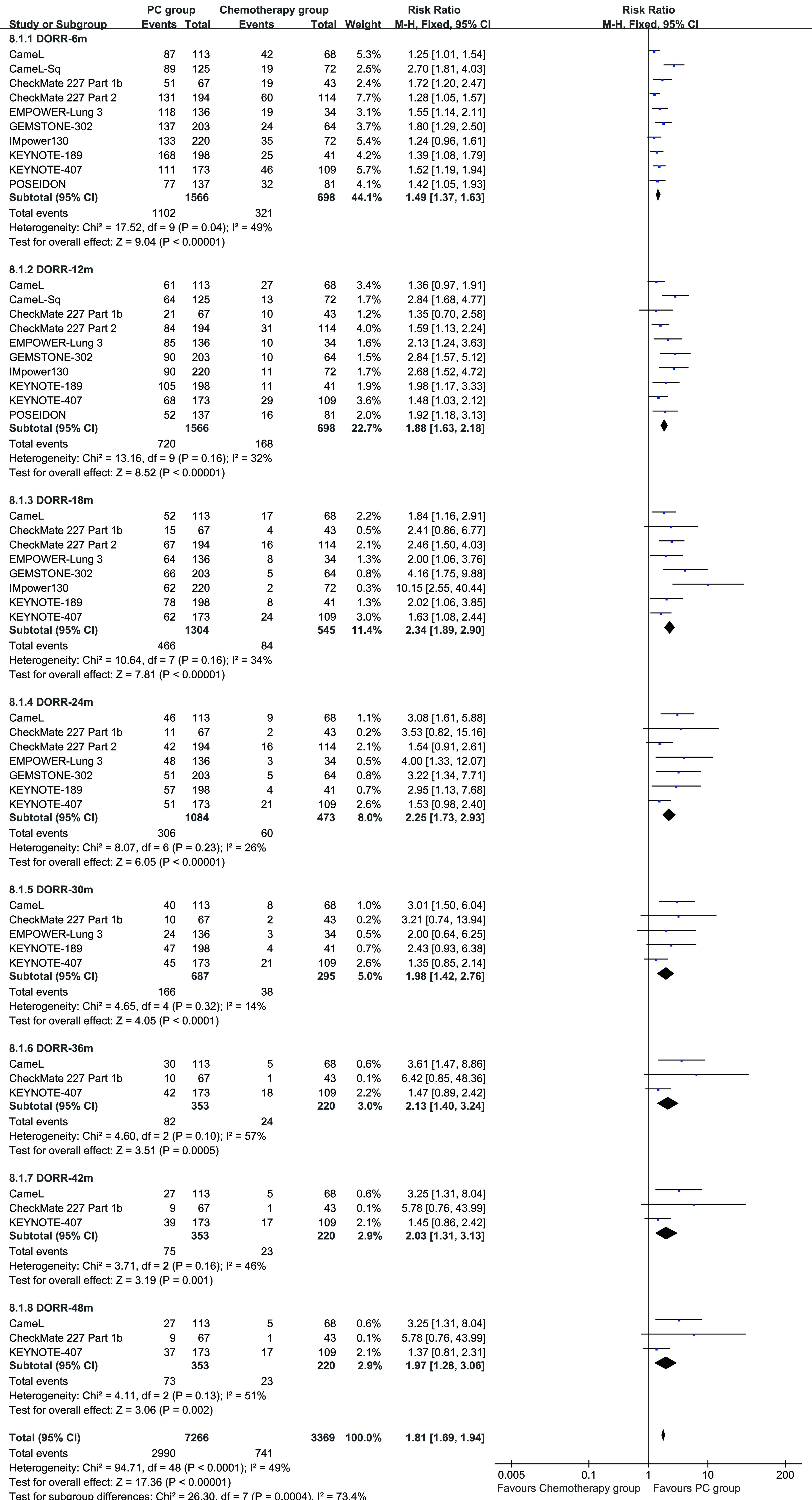

Supplement: Supplementary Figure 4 — Forest plots of DORR at 6–48 months associated with PC versus chemotherapy. [file Image4.tif]

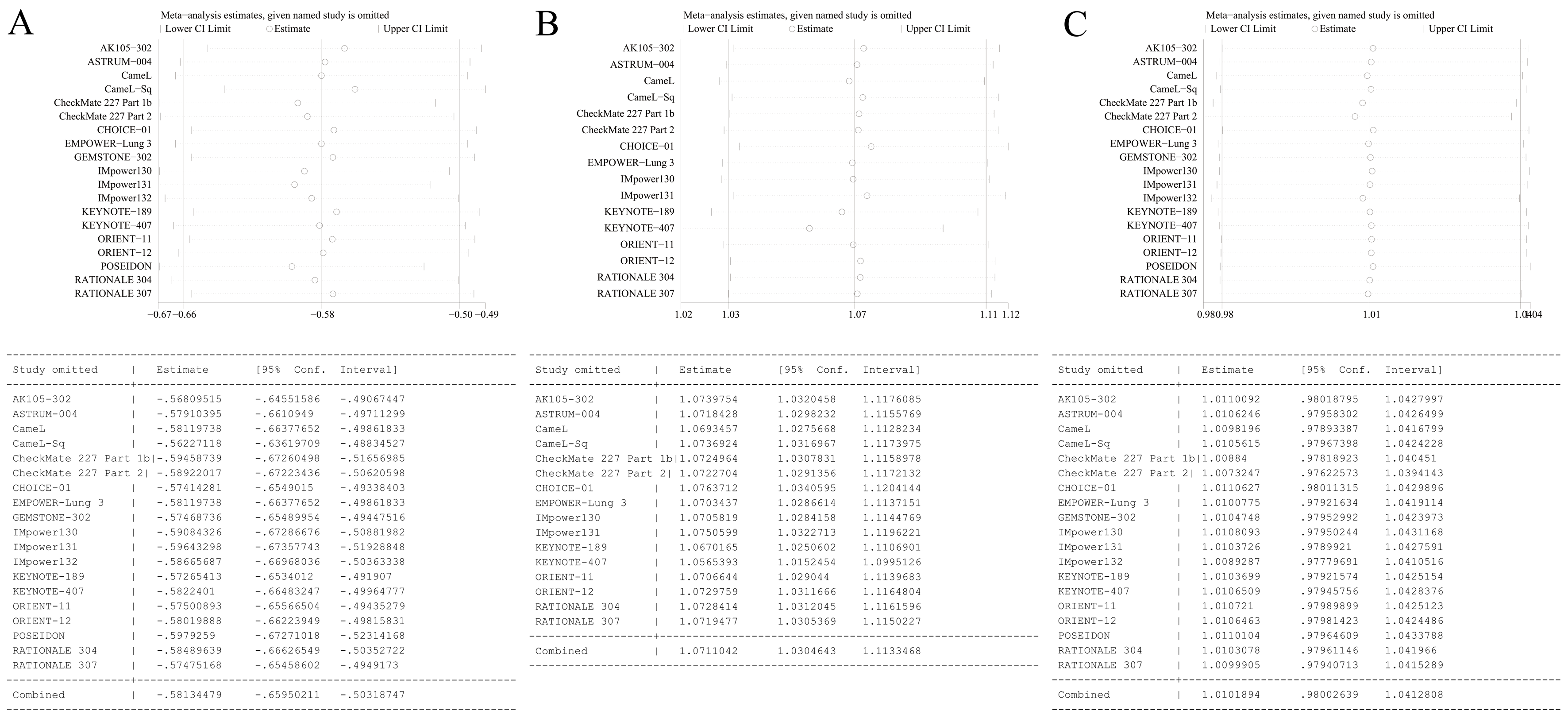

Supplement: Supplementary Figure 6 — Sensitivity analysis of PFS (A), DCR (B), and total TEAEs (C). [file Image6.tif]

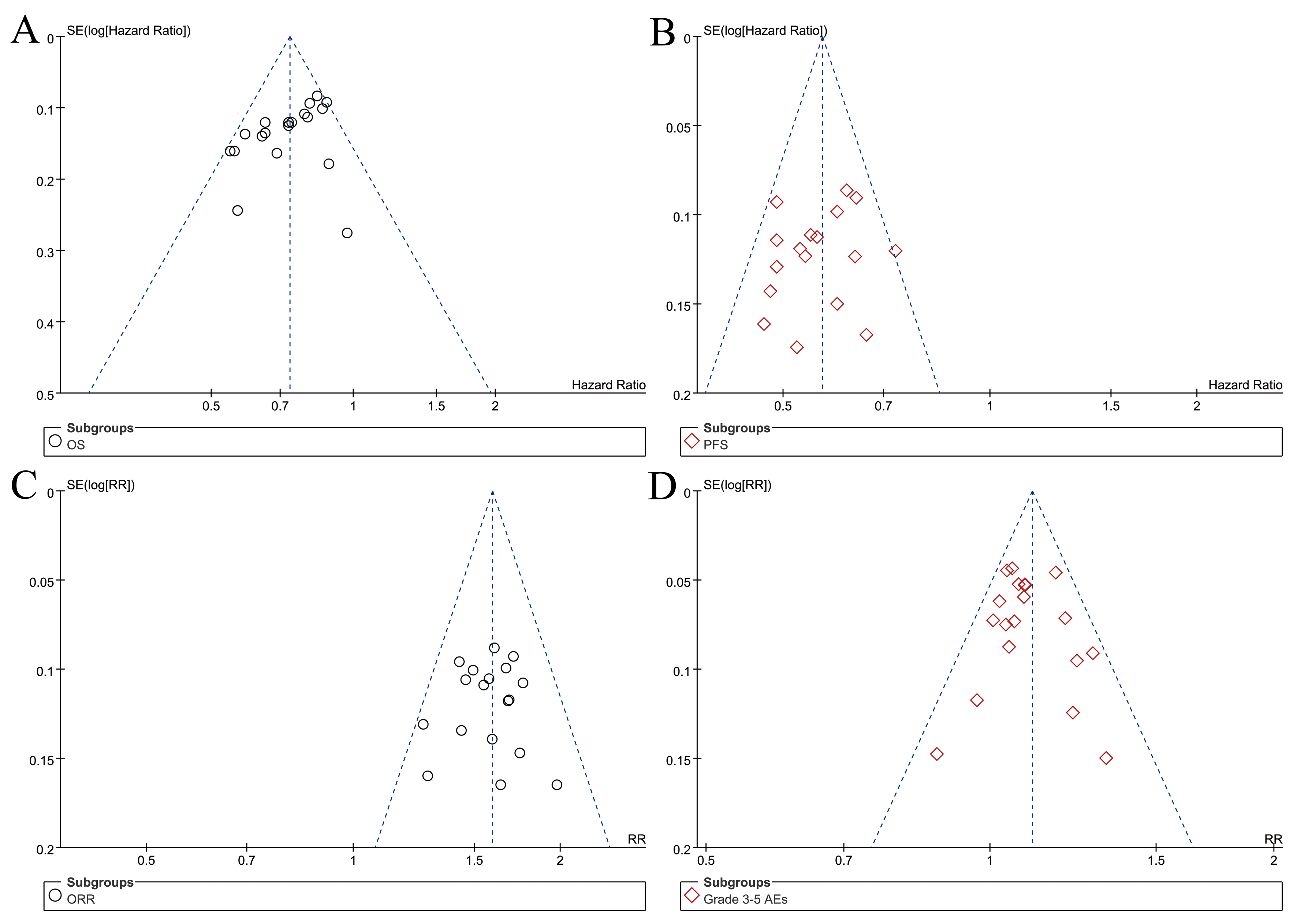

Supplement: Supplementary Figure 7 — Funnel plots of OS (A), PFS (B), ORR (C), and grade 3–5 TEAEs (D). [file Image7.tif]

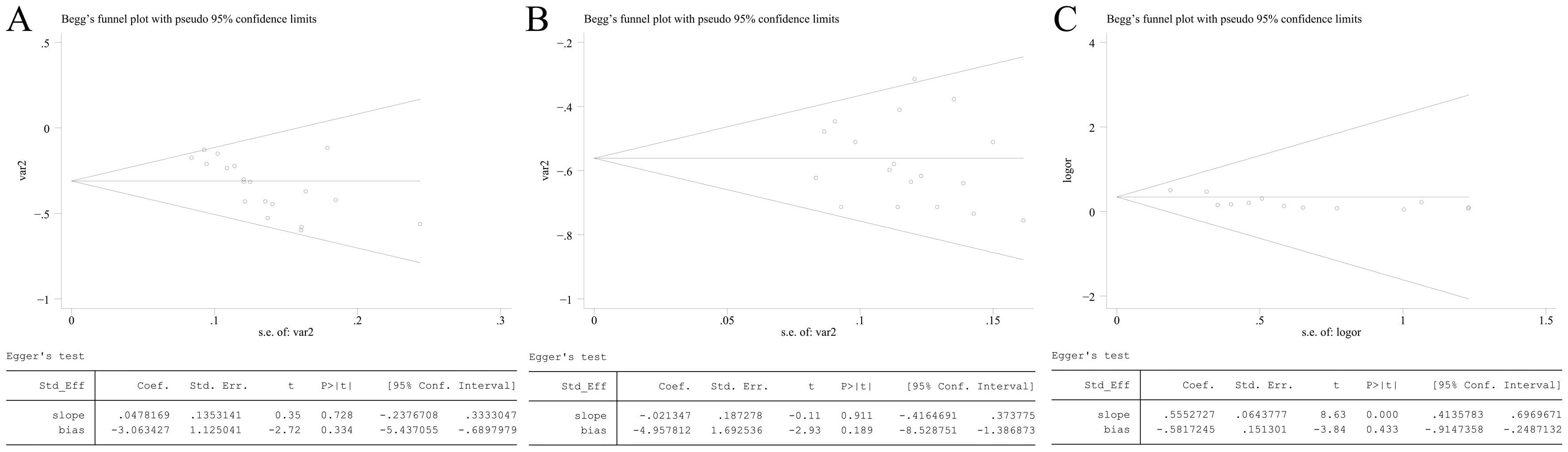

Supplement: Supplementary Figure 8 — Egger’s and Begg’s tests of OS (A), PFS (B), and grade TEAEs (C). [file Image8.tif]
