# Supplementary material for: The benefits and risks of adding PD-1/PD-L1 inhibitors to chemotherapy for stage IIIb-IV non-small-cell lung cancer: an updated meta-analysis based on phase 3 randomized controlled trials
Source: Front Oncol. 2025 Sep 11;15:1590017. doi: 10.3389/fonc.2025.1590017 (PMC12460147; doi:10.3389/fonc.2025.1590017)
Supplement: Supplementary file 9 [file Table1.docx]

**Table S1** Search strategy.

| **PubMed**  The database was searched on February 13, 2025, n=789.  Search Strategy:  **#1**  **Search: Nivolumab[Title/Abstract] OR Pembrolizumab[Title/Abstract] OR Toripalimab[Title/Abstract] OR Sintilimab[Title/Abstract] OR Camrelizumab[Title/Abstract] OR Tislelizumab[Title/Abstract] OR Penpulimab[Title/Abstract] OR Zimberelimab[Title/Abstract] OR Serplulimab[Title/Abstract] OR Durvalumab[Title/Abstract] OR Atezolizumab[Title/Abstract] OR Envolizumab[Title/Abstract] OR Sugemalimab[Title/Abstract] OR Adebrelimab[Title/Abstract] OR Cemiplimab[Title/Abstract] OR Dostarlimab[Title/Abstract] OR Retifanlimab[Title/Abstract] OR Envafolimab[Title/Abstract] OR Suptavumab[Title/Abstract] OR Cadonilimab[Title/Abstract] OR Dostarlimab[Title/Abstract] OR Retifanlimab[Title/Abstract] OR Socazolimab[Title/Abstract] Sort by: Most Recent n = 24174**  **#2 Lung cancer[Title/Abstract] OR NSCLC[Title/Abstract] OR Lung adenocarcinoma[Title/Abstract] OR Lung squamous cell carcinoma[Title/Abstract] Sort by: Most Recent n = 245359**  **#3 Randomized[Title/Abstract]) OR Randomly[Title/Abstract] OR Randomised[Title/Abstract] n = 1224392**  **#1 and #2 and #3 n = 789** |
| --- |
| **Web of Science**  The database was searched on February 13, 2025, n=826.  Search Strategy:  Nivolumab OR Pembrolizumab OR Toripalimab OR Sintilimab OR Camrelizumab OR Tislelizumab OR Penpulimab OR Zimberelimab OR Serplulimab OR Durvalumab OR Atezolizumab OR Envolizumab OR Sugemalimab OR Adebrelimab OR Cemiplimab OR Dostarlimab OR Retifanlimab OR Envafolimab OR Suptavumab OR Cadonilimab OR Dostarlimab OR Retifanlimab OR Socazolimab (Abstract) AND Lung cancer OR NSCLC OR Lung adenocarcinoma OR Lung squamous cell carcinoma(Abstract) AND Randomized OR Randomly OR Randomised (Abstract) and Preprint Citation Index (Exclude - Database) |
| **EMBASE**  The database was searched on February 13, 2025, n=1022.  Search Strategy:  (Nivolumab:ti,ab,kw OR Pembrolizumab:ti,ab,kw OR Toripalimab:ti,ab,kw OR Sintilimab:ti,ab,kw OR Camrelizumab:ti,ab,kw OR Tislelizumab:ti,ab,kw OR Penpulimab:ti,ab,kw OR Zimberelimab:ti,ab,kw OR Serplulimab:ti,ab,kw OR Durvalumab:ti,ab,kw OR Atezolizumab:ti,ab,kw OR Envolizumab:ti,ab,kw OR Sugemalimab:ti,ab,kw OR Adebrelimab:ti,ab,kw OR Cemiplimab:ti,ab,kw OR Dostarlimab:ti,ab,kw OR Retifanlimab:ti,ab,kw OR Envafolimab:ti,ab,kw OR Suptavumab:ti,ab,kw OR Cadonilimab:ti,ab,kw OR Dostarlimab:ti,ab,kw OR Retifanlimab:ti,ab,kw OR Socazolimab:ti,ab,kw) AND (Lung cancer:ti,ab,kw OR NSCLC:ti,ab,kw OR Lung adenocarcinoma:ti,ab,kw OR Lung squamous cell carcinoma:ti,ab,kw) AND **(Randomly**:ti,ab,kw **OR Randomised**:ti,ab,kw **OR**  **Randomized** :ti,ab,kw**)** |
| **Cochrane Library**  The database was searched on February 13, 2025, n=575.  Search Strategy:  (Nivolumab OR Pembrolizumab OR Toripalimab OR Sintilimab OR Camrelizumab OR Tislelizumab OR Penpulimab OR Zimberelimab OR Serplulimab OR Durvalumab OR Atezolizumab OR Envolizumab OR Sugemalimab OR Adebrelimab OR Cemiplimab OR Dostarlimab OR Retifanlimab OR Envafolimab OR Suptavumab OR Cadonilimab OR Dostarlimab OR Retifanlimab OR Socazolimab**)** in Title Abstract Keyword AND (Lung cancer OR NSCLC OR Lung adenocarcinoma OR Lung squamous cell carcinoma**)** in Title Abstract Keyword AND (**Randomized OR Randomly OR Randomised)** in Title Abstract Keyword - (Word variations have been searched) |
| **ScienceDirect**  The database was searched on February 13, 2025, n=2015.  Search Strategy:  Title, abstract, keywords: ((“Nivolumab” OR “Pembrolizumab” OR “Toripalimab” OR “Sintilimab” OR “Camrelizumab” OR “Tislelizumab” OR “Penpulimab” OR “Zimberelimab” OR “Serplulimab” OR “Durvalumab” OR “Atezolizumab” OR “Envolizumab” OR “Sugemalimab” OR “Adebrelimab” OR “Cemiplimab” OR “Dostarlimab” OR “Retifanlimab” OR “Envafolimab” OR “Suptavumab” OR “Cadonilimab” OR “Dostarlimab” OR “Retifanlimab” OR “Socazolimab”) AND (“Lung cancer” OR “NSCLC” OR “Lung adenocarcinoma” OR “Lung squamous cell carcinoma”) AND (“**Randomized**” **OR Randomly**” **OR** “**Randomised**”)) |
| **Scopus**  The database was searched on February 13, 2025, n=1323.  Search Strategy:  (TITLE-ABS-KEY (Nivolumab OR Pembrolizumab OR Toripalimab OR Sintilimab OR Camrelizumab OR Tislelizumab OR Penpulimab OR Zimberelimab OR Serplulimab OR Durvalumab OR Atezolizumab OR Envolizumab OR Sugemalimab OR Adebrelimab OR Cemiplimab OR Dostarlimab OR Retifanlimab OR Envafolimab OR Suptavumab OR Cadonilimab OR Dostarlimab OR Retifanlimab OR Socazolimab) AND TITLE-ABS-KEY (Lung cancer OR NSCLC OR Lung adenocarcinoma OR Lung squamous cell carcinoma) AND TITLE-ABS-KEY (Randomized OR Randomly OR Randomised)) |

**Note:** The combined text and medical subject heading (MeSH) terms used were: “**PD-1/PD-L1 inhibitors**”, “Lung cancer”, and “**Randomized**”.
