# Supplementary material for: The benefits and risks of adding PD-1/PD-L1 inhibitors to chemotherapy for stage IIIb-IV non-small-cell lung cancer: an updated meta-analysis based on phase 3 randomized controlled trials
Source: Front Oncol. 2025 Sep 11;15:1590017. doi: 10.3389/fonc.2025.1590017 (PMC12460147; doi:10.3389/fonc.2025.1590017)
Supplement: Supplementary file 10 [file Table2.doc]

**Table S2 Methodological quality assessments (Jadad scale) of the included studies.**

| **Study** | **Registration No.** | **Randomization** | **Concealment of allocation** | **Double blinding** | **Withdrawals and dropouts** | **Quality (score)** |
| --- | --- | --- | --- | --- | --- | --- |
| AK105-302 [4] | NCT03866993 | ** | ** | * | * | 7 |
| ASTRUM-004 [5] | NCT04033354 | ** | ** | * | * | 7 |
| CameL [17-19] | NCT03134872 | ** | ** | * | * | 7 |
| CameL-Sq [20] | NCT03668496 | ** | ** | * | * | 7 |
| CheckMate 227 Part 1b [21-26] | NCT02477826 | ** | ** | * | * | 7 |
| CheckMate 227 Part 2 [6] | NCT02477826 | ** | ** | * | * | 7 |
| CHOICE-01 [7,27] | NCT03856411 | ** | ** | * | * | 7 |
| EMPOWER-Lung 3 [8,28,29] | NCT03409614 | ** | ** | * | * | 7 |
| GEMSTONE-302 [30,31] | NCT03789604 | ** | ** | * | * | 7 |
| IMpower130 [32] | NCT02367781 | ** | ** | * | * | 7 |
| IMpower131 [33] | NCT02367794 | ** | ** | * | * | 7 |
| IMpower132 [34-35] | NCT02657434 | ** | ** | * | * | 7 |
| KEYNOTE-189 [36-42] | NCT02578680 | ** | ** | * | * | 7 |
| KEYNOTE-407 [43-48] | NCT02775435 | ** | ** | * | * | 7 |
| ORIENT-11 [49-51] | NCT03607539 | ** | ** | * | * | 7 |
| ORIENT-12 [52] | NCT03629925 | ** | ** | * | * | 7 |
| POSEIDON [9,53] | NCT03164616 | ** | ** | * | * | 7 |
| RATIONALE 304 [54,55] | NCT03663205 | ** | ** | * | * | 7 |
| RATIONALE 307 [55,57] | NCT03594747 | ** | ** | * | * | 7 |
