# Supplementary material for: The benefits and risks of adding PD-1/PD-L1 inhibitors to chemotherapy for stage IIIb-IV non-small-cell lung cancer: an updated meta-analysis based on phase 3 randomized controlled trials
Source: Front Oncol. 2025 Sep 11;15:1590017. doi: 10.3389/fonc.2025.1590017 (PMC12460147; doi:10.3389/fonc.2025.1590017)
Supplement: Supplementary file 11 [file Table3.doc]

**Table S3** GRADE quality assessment by therapeutic strategy and study design for the outcomes.

| **Outcomes** | **No. of Participants** | | **Differences (95%CI^) a^** | **Quality Assessment** | | | | | **Quality** |
| --- | --- | --- | --- | --- | --- | --- | --- | --- | --- |
|  | **PC** | **Chemotherapy** |  | **Risk of Bias^b^** | **Inconsistency** | **Indirectness** | **Imprecision** | **Publication Bias^c^** |  |
| **Survival** |  |  |  |  |  |  |  |  |  |
| OS | 9335 | | 0.73 [0.69, 0.77] | Low | No inconsistency | No indirectness | No imprecision | Unlikely | High |
| PFS | 9335 | | 0.56 [0.52, 0.60] | Low | Serious (-1) | No indirectness | No imprecision | Unlikely | Medium |
| **Survival rate** |  |  |  |  |  |  |  |  |  |
| **OSR** |  |  |  |  |  |  |  |  |  |
| OSR-6m | 4535/5326 | 3185/4009 | 1.07 [1.05, 1.09] | Low | No inconsistency | No indirectness | No imprecision | Unlikely | High |
| OSR-12m | 3577/5326 | 2306/4009 | 1.16 [1.12, 1.20] | Low | No inconsistency | No indirectness | No imprecision | Unlikely | High |
| OSR-18m | 2754/5147 | 1611/3831 | 1.25 [1.20, 1.31] | Low | No inconsistency | No indirectness | No imprecision | Unlikely | High |
| OSR-24m | 2043/4804 | 1103/3599 | 1.36 [1.29, 1.45] | Low | No inconsistency | No indirectness | No imprecision | Unlikely | High |
| OSR-30m | 1104/3197 | 556/2507 | 1.54 [1.41, 1.68] | Low | No inconsistency | No indirectness | No imprecision | Unlikely | High |
| OSR-36m | 756/2710 | 415/2178 | 1.42 [1.27, 1.58] | Low | No inconsistency | No indirectness | No imprecision | Unlikely | High |
| OSR-42m | 336/1408 | 180/1217 | 1.60 [1.35, 1.88] | Low | No inconsistency | No indirectness | No imprecision | Unlikely | High |
| OSR-48m | 296/1408 | 164/1217 | 1.55 [1.30, 1.84] | Low | No inconsistency | No indirectness | No imprecision | Unlikely | High |
| OSR-54m | 272/1408 | 133/1217 | 1.76 [1.45, 2.13] | Low | No inconsistency | No indirectness | No imprecision | Unlikely | High |
| OSR-60m | 247/1408 | 118/1217 | 1.79 [1.46, 2.20] | Low | No inconsistency | No indirectness | No imprecision | Unlikely | High |
| **PFSR** |  |  |  |  |  |  |  |  |  |
| PFSR-6m | 3234/5326 | 1640/4009 | 1.48 [1.37, 1.61] | Low | Serious (-1) | No indirectness | No imprecision | Unlikely | Medium |
| PFSR-12m | 1827/5326 | 586/4009 | 2.36 [2.06, 2.70] | Low | Serious (-1) | No indirectness | No imprecision | Unlikely | Medium |
| PFSR-18m | 1265/5147 | 314/3831 | 3.00 [2.66, 3.38] | Low | No inconsistency | No indirectness | No imprecision | Unlikely | High |
| PFSR-24m | 535/3037 | 137/2311 | 3.10 [2.20, 4.37] | Low | Serious (-1) | No indirectness | No imprecision | Unlikely | Medium |
| PFSR-30m | 228/1382 | 51/1034 | 3.68 [2.28, 5.92] | Low | Serious (-1) | No indirectness | No imprecision | Unlikely | Medium |
| PFSR-36m | 96/660 | 33/674 | 2.95 [2.02, 4.31] | Low | No inconsistency | No indirectness | No imprecision | Unlikely | High |
| PFSR-42m | 85/660 | 29/674 | 2.97 [1.98, 4.46] | Low | No inconsistency | No indirectness | No imprecision | Unlikely | High |
| PFSR-48m | 80/660 | 20/674 | 4.68 [1.64, 13.34] | Low | Serious (-1) | No indirectness | No imprecision | Unlikely | Medium |
| PFSR-54m | 77/660 | 17/674 | 4.49 [2.71, 7.45] | Low | No inconsistency | No indirectness | No imprecision | Unlikely | High |
| PFSR-60m | 73/660 | 16/674 | 4.52 [2.68, 7.61] | Low | No inconsistency | No indirectness | No imprecision | Unlikely | High |
| **Subgroup analysis of OS** |  |  |  |  |  |  |  |  |  |
| Total | 9335 | | 0.73 [0.69, 0.77] | Low | No inconsistency | No indirectness | No imprecision | Unlikely | High |
| Age - < 65 years | 4066 | | 0.68 [0.59, 0.77] | Low | Serious (-1) | No indirectness | No imprecision | Unlikely | Medium |
| Age - > 65 years | 3291 | | 0.79 [0.72, 0.87] | Low | No inconsistency | No indirectness | No imprecision | Unlikely | High |
| Sex - Female | 1849 | | 0.69 [0.61, 0.78] | Low | No inconsistency | No indirectness | No imprecision | Unlikely | High |
| Sex - Male | 5159 | | 0.76 [0.71, 0.82] | Low | No inconsistency | No indirectness | No imprecision | Unlikely | High |
| Race - Asia | 4136 | | 0.69 [0.64, 0.76] | Low | No inconsistency | No indirectness | No imprecision | Unlikely | High |
| Race - White | 1380 | | 0.78 [0.63, 0.97] | Low | Serious (-1) | No indirectness | No imprecision | Unlikely | Medium |
| ECOG PS - 0 | 2251 | | 0.70 [0.63, 0.79] | Low | No inconsistency | No indirectness | No imprecision | Unlikely | High |
| ECOG PS - 1 | 5191 | | 0.75 [0.70, 0.80] | Low | No inconsistency | No indirectness | No imprecision | Unlikely | High |
| Smoking status - Current/former | 4866 | | 0.67 [0.60, 0.76] | Low | Serious (-1) | No indirectness | No imprecision | Unlikely | Medium |
| Smoking status - Never | 1125 | | 0.82 [0.70, 0.96] | Low | No inconsistency | No indirectness | No imprecision | Unlikely | High |
| Pathological type - Squamous | 4319 | | 0.72 [0.67, 0.79] | Low | No inconsistency | No indirectness | No imprecision | Unlikely | High |
| Pathological type - Non-squamous | 5054 | | 0.73 [0.68, 0.79] | Low | No inconsistency | No indirectness | No imprecision | Unlikely | High |
| Stage - Stage III | 396 | | 0.70 [0.52, 0.96] | Low | No inconsistency | No indirectness | No imprecision | Unlikely | High |
| Stage - Stage IV | 7392 | | 0.71 [0.65, 0.78] | Low | Serious (-1) | No indirectness | No imprecision | Unlikely | Medium |
| Brain metastases - Yes | 363 | | 0.61 [0.47, 0.79] | Low | No inconsistency | No indirectness | No imprecision | Unlikely | High |
| Brain metastases - No | 3079 | | 0.70 [0.65, 0.77] | Low | No inconsistency | No indirectness | No imprecision | Unlikely | High |
| Liver metastases - Yes | 673 | | 0.84 [0.70, 1.00] | Low | No inconsistency | No indirectness | No imprecision | Unlikely | High |
| Liver metastases - No | 3012 | | 0.78 [0.68, 0.91] | Low | Serious (-1) | No indirectness | No imprecision | Unlikely | Medium |
| PD-L1 CPS - <1% | 3393 | | 0.80 [0.73, 0.87] | Low | No inconsistency | No indirectness | No imprecision | Unlikely | High |
| PD-L1 CPS - >1% | 2711 | | 0.68 [0.61, 0.75] | Low | No inconsistency | No indirectness | No imprecision | Unlikely | High |
| PD-L1 CPS - 1%-49% | 2030 | | 0.70 [0.62, 0.80] | Low | No inconsistency | No indirectness | No imprecision | Unlikely | High |
| PD-L1 CPS - >50% | 1416 | | 0.61 [0.52, 0.71] | Low | No inconsistency | No indirectness | No imprecision | Unlikely | High |
| PD-1/PD-L1 inhibitors type - Penpulimab | 350 | | 0.55 [0.40, 0.75] | Low | No inconsistency | No indirectness | No imprecision | Unlikely | High |
| PD-1/PD-L1 inhibitors type - Sugemalimab | 1016 | | 0.67 [0.56, 0.79] | Low | No inconsistency | No indirectness | No imprecision | Unlikely | High |
| PD-1/PD-L1 inhibitors type - Camrelizumab | 801 | | 0.67 [0.55, 0.81] | Low | No inconsistency | No indirectness | No imprecision | Unlikely | High |
| PD-1/PD-L1 inhibitors type - Nivolumab | 1118 | | 0.81 [0.70, 0.93] | Low | No inconsistency | No indirectness | No imprecision | Unlikely | High |
| PD-1/PD-L1 inhibitors type - Toripalimab | 465 | | 0.73 [0.57, 0.93] | Low | No inconsistency | No indirectness | No imprecision | Unlikely | High |
| PD-1/PD-L1 inhibitors type - Cemiplimab | 466 | | 0.65 [0.51, 0.82] | Low | No inconsistency | No indirectness | No imprecision | Unlikely | High |
| PD-1/PD-L1 inhibitors type - Atezolizumab | 1940 | | 0.85 [0.76, 0.95] | Low | No inconsistency | No indirectness | No imprecision | Unlikely | High |
| PD-1/PD-L1 inhibitors type - Pembrolizumab | 1175 | | 0.59 [0.50, 0.69] | Low | No inconsistency | No indirectness | No imprecision | Unlikely | High |
| PD-1/PD-L1 inhibitors type - Sintilimab | 794 | | 0.63 [0.50, 0.79] | Low | No inconsistency | No indirectness | No imprecision | Unlikely | High |
| PD-1/PD-L1 inhibitors type - Durvalumab | 675 | | 0.84 [0.71, 0.99] | Low | No inconsistency | No indirectness | No imprecision | Unlikely | High |
| PD-1/PD-L1 inhibitors type - Tislelizumab | 575 | | 0.77 [0.61, 0.98] | Low | No inconsistency | No indirectness | No imprecision | Unlikely | High |
| Platinum chemotherapy type - Cisplatin | 501 | | 0.65 [0.44, 0.95] | Low | Serious (-1) | No indirectness | No imprecision | Unlikely | Medium |
| Platinum chemotherapy type - Carboplatin | 5851 | | 0.72 [0.67, 0.77] | Low | No inconsistency | No indirectness | No imprecision | Unlikely | High |
| **Subgroup analysis of PFS** |  |  |  |  |  |  |  |  |  |
| Total | 9335 | | 0.56 [0.52, 0.60] | Low | Serious (-1) | No indirectness | No imprecision | Unlikely | Medium |
| Age - < 65 years | 4568 | | 0.52 [0.46, 0.59] | Low | Serious (-1) | No indirectness | No imprecision | Unlikely | Medium |
| Age - > 65 years | 3648 | | 0.59 [0.55, 0.64] | Low | No inconsistency | No indirectness | No imprecision | Unlikely | High |
| Sex - Female | 1819 | | 0.58 [0.52, 0.65] | Low | No inconsistency | No indirectness | No imprecision | Unlikely | High |
| Sex - Male | 6048 | | 0.55 [0.49, 0.60] | Low | Serious (-1) | No indirectness | No imprecision | Unlikely | Medium |
| Race - Asia | 4415 | | 0.50 [0.47, 0.54] | Low | No inconsistency | No indirectness | No imprecision | Unlikely | High |
| Race - White | 1558 | | 0.66 [0.59, 0.73] | Low | No inconsistency | No indirectness | No imprecision | Unlikely | High |
| ECOG PS - 0 | 2300 | | 0.53 [0.48, 0.59] | Low | No inconsistency | No indirectness | No imprecision | Unlikely | High |
| ECOG PS - 1 | 5902 | | 0.56 [0.51, 0.61] | Low | Serious (-1) | No indirectness | No imprecision | Unlikely | Medium |
| Smoking status - Current/former | 4984 | | 0.51 [0.45, 0.58] | Low | Serious (-1) | No indirectness | No imprecision | Unlikely | Medium |
| Smoking status - Never | 1282 | | 0.62 [0.54, 0.71] | Low | No inconsistency | No indirectness | No imprecision | Unlikely | High |
| Pathological type - Squamous | 4279 | | 0.53 [0.47, 0.60] | Low | Serious (-1) | No indirectness | No imprecision | Unlikely | Medium |
| Pathological type - Non-squamous | 5053 | | 0.59 [0.55, 0.63] | Low | No inconsistency | No indirectness | No imprecision | Unlikely | High |
| Stage - Stage III | 713 | | 0.42 [0.35, 0.51] | Low | No inconsistency | No indirectness | No imprecision | Unlikely | High |
| Stage - Stage IV | 8210 | | 0.57 [0.52, 0.61] | Low | Serious (-1) | No indirectness | No imprecision | Unlikely | Medium |
| Brain metastases - Yes | 319 | | 0.41 [0.31, 0.55] | Low | No inconsistency | No indirectness | No imprecision | Unlikely | High |
| Brain metastases - No | 2707 | | 0.52 [0.47, 0.57] | Low | No inconsistency | No indirectness | No imprecision | Unlikely | High |
| Liver metastases - Yes | 675 | | 0.71 [0.60, 0.85] | Low | No inconsistency | No indirectness | No imprecision | Unlikely | High |
| Liver metastases - No | 3869 | | 0.56 [0.52, 0.60] | Low | No inconsistency | No indirectness | No imprecision | Unlikely | High |
| PD-L1 CPS - <1% | 3706 | | 0.69 [0.63, 0.75] | Low | No inconsistency | No indirectness | No imprecision | Unlikely | High |
| PD-L1 CPS - >1% | 2976 | | 0.48 [0.44, 0.52] | Low | No inconsistency | No indirectness | No imprecision | Unlikely | High |
| PD-L1 CPS - 1%-49% | 2282 | | 0.55 [0.50, 0.62] | Low | No inconsistency | No indirectness | No imprecision | Unlikely | High |
| PD-L1 CPS - >50% | 1757 | | 0.45 [0.39, 0.50] | Low | No inconsistency | No indirectness | No imprecision | Unlikely | High |
| PD-1/PD-L1 inhibitors type - Penpulimab | 350 | | 0.43 [0.33, 0.56] | Low | No inconsistency | No indirectness | No imprecision | Unlikely | High |
| PD-1/PD-L1 inhibitors type - Sugemalimab | 1016 | | 0.50 [0.43, 0.59] | Low | No inconsistency | No indirectness | No imprecision | Unlikely | High |
| PD-1/PD-L1 inhibitors type - Camrelizumab | 801 | | 0.47 [0.35, 0.64] | Low | Serious (-1) | No indirectness | No imprecision | Unlikely | Medium |
| PD-1/PD-L1 inhibitors type - Nivolumab | 1118 | | 0.66 [0.57, 0.75] | Low | No inconsistency | No indirectness | No imprecision | Unlikely | High |
| PD-1/PD-L1 inhibitors type - Toripalimab | 465 | | 0.49 [0.39, 0.61] | Low | No inconsistency | No indirectness | No imprecision | Unlikely | High |
| PD-1/PD-L1 inhibitors type - Cemiplimab | 466 | | 0.55 [0.44, 0.68] | Low | No inconsistency | No indirectness | No imprecision | Unlikely | High |
| PD-1/PD-L1 inhibitors type - Atezolizumab | 1940 | | 0.65 [0.59, 0.72] | Low | No inconsistency | No indirectness | No imprecision | Unlikely | High |
| PD-1/PD-L1 inhibitors type - Pembrolizumab | 1175 | | 0.52 [0.45, 0.60] | Low | No inconsistency | No indirectness | No imprecision | Unlikely | High |
| PD-1/PD-L1 inhibitors type - Sintilimab | 754 | | 0.52 [0.43, 0.61] | Low | No inconsistency | No indirectness | No imprecision | Unlikely | High |
| PD-1/PD-L1 inhibitors type - Durvalumab | 675 | | 0.74 [0.62, 0.89] | Low | No inconsistency | No indirectness | No imprecision | Unlikely | High |
| PD-1/PD-L1 inhibitors type - Tislelizumab | 575 | | 0.54 [0.43, 0.66] | Low | No inconsistency | No indirectness | No imprecision | Unlikely | High |
| Platinum chemotherapy type - Cisplatin | 636 | | 0.55 [0.46, 0.66] | Low | No inconsistency | No indirectness | No imprecision | Unlikely | High |
| Platinum chemotherapy type - Carboplatin | 6073 | | 0.54 [0.50, 0.57] | Low | No inconsistency | No indirectness | No imprecision | Unlikely | High |
| **Responses** |  |  |  |  |  |  |  |  |  |
| ORR | 2851/5326 | 1348/4009 | 1.59 [1.51, 1.67] | Low | No inconsistency | No indirectness | No imprecision | Unlikely | High |
| DCR | 3737/4376 | 2433/3227 | 1.12 [1.07, 1.18] | Low | Serious (-1) | No indirectness | No imprecision | Unlikely | Medium |
| CR | 127/4668 | 45/3513 | 2.30 [1.64, 3.23] | Low | No inconsistency | No indirectness | No imprecision | Unlikely | High |
| PR | 2384/4668 | 1160/3513 | 1.55 [1.47, 1.64] | Low | No inconsistency | No indirectness | No imprecision | Unlikely | High |
| SD | 1354/4376 | 1382/3227 | 0.71 [0.65, 0.78] | Low | Serious (-1) | No indirectness | No imprecision | Unlikely | Medium |
| PD | 344/4376 | 454/3227 | 0.55 [0.48, 0.63] | Low | No inconsistency | No indirectness | No imprecision | Unlikely | High |
| DOR | 3903 | 2842 | 0.50 [0.45, 0.54] | Low | No inconsistency | No indirectness | No imprecision | Unlikely | High |
| **DORR** |  |  |  |  |  |  |  |  |  |
| DORR-6m | 1102/1566 | 321/698 | 1.49 [1.37, 1.63] | Low | No inconsistency | No indirectness | No imprecision | Unlikely | High |
| DORR-12m | 720/1566 | 168/698 | 1.88 [1.63, 2.18] | Low | No inconsistency | No indirectness | No imprecision | Unlikely | High |
| DORR-18m | 466/1304 | 84/545 | 2.34 [1.89, 2.90] | Low | No inconsistency | No indirectness | No imprecision | Unlikely | High |
| DORR-24m | 306/1084 | 60/473 | 2.25 [1.73, 2.93] | Low | No inconsistency | No indirectness | No imprecision | Unlikely | High |
| DORR-30m | 166/687 | 38/295 | 1.98 [1.42, 2.76] | Low | No inconsistency | No indirectness | No imprecision | Unlikely | High |
| DORR-36m | 82/353 | 24/220 | 2.13 [1.40, 3.24] | Low | No inconsistency | No indirectness | No imprecision | Unlikely | High |
| DORR-42m | 75/353 | 23/220 | 2.03 [1.31, 3.13] | Low | No inconsistency | No indirectness | No imprecision | Unlikely | High |
| DORR-48m | 73/353 | 23/220 | 1.97 [1.28, 3.06] | Low | No inconsistency | No indirectness | No imprecision | Unlikely | High |
| **Safety summary** |  |  |  |  |  |  |  |  |  |
| Total TEAEs | 5178/5326 | 3793/4009 | 1.01 [1.00, 1.02] | Low | Serious (-1) | No indirectness | No imprecision | Unlikely | Medium |
| Grade 3-5 TEAEs | 3606/5326 | 2404/4009 | 1.10 [1.05, 1.15] | Low | Serious (-2) | No indirectness | No imprecision | Unlikely | Medium |
| Serious TEAEs | 1481/3504 | 774/2681 | 1.43 [1.26, 1.62] | Low | Serious (-3) | No indirectness | No imprecision | Unlikely | Medium |
| TEAEs leading to discontinuation | 1081/5326 | 479/4009 | 1.65 [1.40, 1.94] | Low | Serious (-4) | No indirectness | No imprecision | Unlikely | Medium |
| TEAEs leading to death | 375/4949 | 235/3631 | 1.20 [1.02, 1.40] | Low | No inconsistency | No indirectness | No imprecision | Unlikely | High |
| TRAEs | 3969/4349 | 3025/3433 | 1.03 [1.01, 1.06] | Low | Serious (-4) | No indirectness | No imprecision | Unlikely | Medium |
| Grade 3-5 TRAEs | 2384/4349 | 1660/3433 | 1.14 [1.06, 1.23] | Low | Serious (-5) | No indirectness | No imprecision | Unlikely | Medium |
| Serious TRAEs | 725/2852 | 375/2293 | 1.55 [1.27, 1.90] | Low | Serious (-6) | No indirectness | No imprecision | Unlikely | Medium |
| TRAEs leading to discontinuation | 450/3143 | 162/2458 | 2.17 [1.83, 2.58] | Low | No inconsistency | No indirectness | No imprecision | Unlikely | High |
| TRAEs leading to death | 100/4229 | 52/3312 | 1.58 [1.13, 2.21] | Low | No inconsistency | No indirectness | No imprecision | Unlikely | High |
| irAEs | 1312/3719 | 404/2548 | 2.59 [1.94, 3.47] | Low | Serious (-6) | No indirectness | No imprecision | Unlikely | Medium |
| Grade 3-5 irAEs | 341/3719 | 77/2548 | 3.04 [2.38, 3.87] | Low | No inconsistency | No indirectness | No imprecision | Unlikely | High |
| Serious irAEs | 31/513 | 6/512 | 5.16 [2.17, 12.25] | Low | No inconsistency | No indirectness | No imprecision | Unlikely | High |
| irAEs leading to discontinuation | 20/513 | 3/512 | 6.65 [1.99, 22.25] | Low | No inconsistency | No indirectness | No imprecision | Unlikely | High |
| irAEs leading to death | 11/1957 | 1/1545 | 3.19 [0.89, 11.41] | Low | No inconsistency | No indirectness | No imprecision | Unlikely | High |
| **TEAEs** |  |  |  |  |  |  |  |  |  |
| **Any grade** |  |  |  |  |  |  |  |  |  |
| Anemia | 2933/5034 | 2076/3723 | 1.03 [0.99, 1.06] | Low | No inconsistency | No indirectness | No imprecision | Unlikely | High |
| Neutrophil count decreased | 1372/3502 | 1067/2815 | 1.04 [0.98, 1.09] | Low | No inconsistency | No indirectness | No imprecision | Unlikely | High |
| White blood cell decreased | 1215/3299 | 914/2446 | 1.05 [0.99, 1.11] | Low | No inconsistency | No indirectness | No imprecision | Unlikely | High |
| Nausea | 1725/5034 | 1169/3723 | 1.07 [1.00, 1.13] | Low | No inconsistency | No indirectness | No imprecision | Unlikely | High |
| Neutropenia | 1340/4016 | 856/2836 | 1.05 [0.98, 1.12] | Low | No inconsistency | No indirectness | No imprecision | Unlikely | High |
| Alopecia | 1001/3265 | 754/2548 | 1.09 [1.02, 1.17] | Low | No inconsistency | No indirectness | No imprecision | Unlikely | High |
| Platelet count decreased | 893/3099 | 628/2254 | 1.10 [1.01, 1.19] | Low | No inconsistency | No indirectness | No imprecision | Unlikely | High |
| Fatigue | 1055/4071 | 632/2890 | 1.12 [1.03, 1.22] | Low | No inconsistency | No indirectness | No imprecision | Unlikely | High |
| Alanine aminotransferase increased | 857/3329 | 515/2211 | 1.11 [1.01, 1.22] | Low | No inconsistency | No indirectness | No imprecision | Unlikely | High |
| Leukopenia | 711/2774 | 401/1785 | 1.06 [0.97, 1.15] | Low | No inconsistency | No indirectness | No imprecision | Unlikely | High |
| Thrombocytopenia | 970/3839 | 588/2650 | 1.07 [0.99, 1.17] | Low | No inconsistency | No indirectness | No imprecision | Unlikely | High |
| Aspartate aminotransferase increased | 811/3329 | 429/2211 | 1.25 [1.13, 1.38] | Low | No inconsistency | No indirectness | No imprecision | Unlikely | High |
| Decreased appetite | 1208/5034 | 828/3723 | 1.09 [1.01, 1.18] | Low | No inconsistency | No indirectness | No imprecision | Unlikely | High |
| Hypoesthesia | 183/846 | 146/671 | 1.19 [0.99, 1.43] | Low | No inconsistency | No indirectness | No imprecision | Unlikely | High |
| Constipation | 1039/4841 | 673/3527 | 1.09 [1.00, 1.19] | Low | No inconsistency | No indirectness | No imprecision | Unlikely | High |
| Cough | 419/2009 | 220/1319 | 1.19 [0.90, 1.58] | Low | Serious (-6) | No indirectness | No imprecision | Unlikely | Medium |
| Diarrhea | 777/4041 | 428/2975 | 1.30 [1.17, 1.45] | Low | No inconsistency | No indirectness | No imprecision | Unlikely | High |
| Vomiting | 876/4657 | 543/3345 | 1.13 [1.02, 1.24] | Low | No inconsistency | No indirectness | No imprecision | Unlikely | High |
| Asthenia | 809/4327 | 587/3281 | 1.05 [0.96, 1.16] | Low | No inconsistency | No indirectness | No imprecision | Unlikely | High |
| Pyrexia | 467/2782 | 237/1892 | 1.31 [1.14, 1.52] | Low | No inconsistency | No indirectness | No imprecision | Unlikely | High |
| Hypomagnesaemia | 133/794 | 65/568 | 1.30 [0.99, 1.72] | Low | No inconsistency | No indirectness | No imprecision | Unlikely | High |
| Pain in extremity | 406/2559 | 211/1531 | 1.27 [0.98, 1.65] | Low | Serious (-6) | No indirectness | No imprecision | Unlikely | Medium |
| Dyspnea | 333/2129 | 166/1207 | 1.13 [0.82, 1.55] | Low | Serious (-6) | No indirectness | No imprecision | Unlikely | Medium |
| Hypoalbuminaemia | 330/2176 | 142/1235 | 1.38 [1.15, 1.66] | Low | No inconsistency | No indirectness | No imprecision | Unlikely | High |
| Rash | 676/4545 | 244/3481 | 2.07 [1.81, 2.38] | Low | No inconsistency | No indirectness | No imprecision | Unlikely | High |
| Arthralgia | 315/2210 | 195/1832 | 1.31 [1.11, 1.55] | Low | No inconsistency | No indirectness | No imprecision | Unlikely | High |
| Edema peripheral | 241/1695 | 98/956 | 1.36 [1.09, 1.69] | Low | No inconsistency | No indirectness | No imprecision | Unlikely | High |
| Peripheral sensory neuropathy | 195/1384 | 116/1003 | 1.29 [1.04, 1.60] | Low | No inconsistency | No indirectness | No imprecision | Unlikely | High |
| Weight decreased | 222/1710 | 143/1043 | 0.95 [0.70, 1.30] | Low | Serious (-6) | No indirectness | No imprecision | Unlikely | Medium |
| Pruritus | 185/1449 | 57/1239 | 3.07 [1.76, 5.36] | Low | Serious (-6) | No indirectness | No imprecision | Unlikely | Medium |
| Headache | 100/789 | 48/565 | 1.27 [0.70, 2.32] | Low | Serious (-6) | No indirectness | No imprecision | Unlikely | Medium |
| Hypokalaemia | 186/1567 | 84/872 | 1.26 [0.99, 1.61] | Low | No inconsistency | No indirectness | No imprecision | Unlikely | High |
| Hyperuricaemia | 57/484 | 36/331 | 1.11 [0.54, 2.28] | Low | Serious (-6) | No indirectness | No imprecision | Unlikely | Medium |
| Hepatic function abnormal | 59/525 | 36/366 | 1.45 [0.98, 2.14] | Low | No inconsistency | No indirectness | No imprecision | Unlikely | High |
| Hypertriglyceridaemia | 55/495 | 33/334 | 1.50 [1.02, 2.23] | Low | No inconsistency | No indirectness | No imprecision | Unlikely | High |
| Hyperglycemia | 151/1392 | 52/697 | 1.45 [1.08, 1.95] | Low | No inconsistency | No indirectness | No imprecision | Unlikely | High |
| Hyponatraemia | 165/1554 | 103/1017 | 1.23 [0.97, 1.56] | Low | No inconsistency | No indirectness | No imprecision | Unlikely | High |
| Hypothyroidism | 274/2663 | 27/1802 | 6.39 [4.33, 9.42] | Low | No inconsistency | No indirectness | No imprecision | Unlikely | High |
| Gamma-glutamyltransferase increased | 134/1344 | 89/965 | 1.22 [0.81, 1.85] | Low | Serious (-6) | No indirectness | No imprecision | Unlikely | Medium |
| Pneumonia | 205/2135 | 114/1424 | 1.25 [1.01, 1.55] | Low | No inconsistency | No indirectness | No imprecision | Unlikely | High |
| Blood creatinine increased | 120/1288 | 36/748 | 2.09 [1.46, 3.01] | Low | No inconsistency | No indirectness | No imprecision | Unlikely | High |
| Hemoptysis | 140/1554 | 84/1017 | 1.24 [0.96, 1.61] | Low | No inconsistency | No indirectness | No imprecision | Unlikely | High |
| Urinary tract infection | 67/771 | 22/387 | 1.54 [0.97, 2.43] | Low | No inconsistency | No indirectness | No imprecision | Unlikely | High |
| Proteinuria | 73/871 | 49/691 | 1.43 [0.71, 2.87] | Low | Serious (-6) | No indirectness | No imprecision | Unlikely | Medium |
| Blood bilirubin increased | 111/1405 | 58/871 | 1.33 [0.83, 2.12] | Low | Serious (-6) | No indirectness | No imprecision | Unlikely | Medium |
| Hyperthyroidism | 60/760 | 4/384 | 7.58 [2.78, 20.64] | Low | No inconsistency | No indirectness | No imprecision | Unlikely | High |
| Malaise | 60/798 | 36/459 | 1.15 [0.78, 1.71] | Low | No inconsistency | No indirectness | No imprecision | Unlikely | High |
| Myalgia | 56/794 | 25/568 | 1.49 [0.92, 2.40] | Low | No inconsistency | No indirectness | No imprecision | Unlikely | High |
| Lymphocyte count decreased | 77/1169 | 45/790 | 1.39 [0.97, 1.99] | Low | No inconsistency | No indirectness | No imprecision | Unlikely | High |
| Hypercholesteraemia | 32/495 | 15/334 | 1.87 [1.03, 3.39] | Low | No inconsistency | No indirectness | No imprecision | Unlikely | High |
| Stomatitis | 43/771 | 12/387 | 1.76 [0.95, 3.24] | Low | No inconsistency | No indirectness | No imprecision | Unlikely | High |
| Blood lactatedehydrogenase increased | 35/632 | 9/313 | 1.93 [0.94, 3.95] | Low | No inconsistency | No indirectness | No imprecision | Unlikely | High |
| Myelosuppression | 29/525 | 14/366 | 1.37 [0.37, 5.12] | Low | Serious (-6) | No indirectness | No imprecision | Unlikely | Medium |
| Upper respiratory tract infection | 40/771 | 12/387 | 1.64 [0.88, 3.03] | Low | No inconsistency | No indirectness | No imprecision | Unlikely | High |
| Blood alkaline phosphatase increased | 56/1083 | 24/541 | 1.16 [0.73, 1.86] | Low | No inconsistency | No indirectness | No imprecision | Unlikely | High |
| Lymphopenia | 39/760 | 23/384 | 0.86 [0.53, 1.39] | Low | No inconsistency | No indirectness | No imprecision | Unlikely | High |
| Abdominal pain upper | 33/771 | 12/387 | 1.89 [0.32, 11.30] | Low | Serious (-6) | No indirectness | No imprecision | Unlikely | Medium |
| Hypertension | 28/771 | 10/387 | 1.41 [0.69, 2.87] | Low | No inconsistency | No indirectness | No imprecision | Unlikely | High |
| Febrile neutropenia | 37/1114 | 15/727 | 1.73 [0.97, 3.10] | Low | No inconsistency | No indirectness | No imprecision | Unlikely | High |
| Interstitial lung disease | 18/563 | 1/386 | 7.75 [1.50, 40.19] | Low | No inconsistency | No indirectness | No imprecision | Unlikely | High |
| Renal failure | 13/771 | 4/387 | 1.51 [0.52, 4.36] | Low | No inconsistency | No indirectness | No imprecision | Unlikely | High |
| **Grade 3-5** |  |  |  |  |  |  |  |  |  |
| Neutrophil count decreased | 834/3502 | 652/2815 | 1.05 [0.96, 1.14] | Low | No inconsistency | No indirectness | No imprecision | Unlikely | High |
| Neutropenia | 814/4016 | 515/2836 | 1.07 [0.97, 1.18] | Low | No inconsistency | No indirectness | No imprecision | Unlikely | High |
| Anemia | 895/5034 | 622/3723 | 1.04 [0.95, 1.14] | Low | No inconsistency | No indirectness | No imprecision | Unlikely | High |
| White blood cell decreased | 418/3299 | 310/2446 | 1.09 [0.95, 1.24] | Low | No inconsistency | No indirectness | No imprecision | Unlikely | High |
| Platelet count decreased | 296/3099 | 195/2254 | 1.19 [1.01, 1.40] | Low | No inconsistency | No indirectness | No imprecision | Unlikely | High |
| Leukopenia | 250/2774 | 146/1785 | 1.02 [0.85, 1.23] | Low | No inconsistency | No indirectness | No imprecision | Unlikely | High |
| Thrombocytopenia | 291/3839 | 165/2650 | 1.17 [0.98, 1.41] | Low | No inconsistency | No indirectness | No imprecision | Unlikely | High |
| Pneumonia | 102/2135 | 61/1424 | 1.15 [0.85, 1.56] | Low | No inconsistency | No indirectness | No imprecision | Unlikely | High |
| Fatigue | 148/4071 | 69/2890 | 1.49 [1.13, 1.98] | Low | No inconsistency | No indirectness | No imprecision | Unlikely | High |
| Myelosuppression | 19/525 | 7/366 | 2.18 [0.96, 4.95] | Low | No inconsistency | No indirectness | No imprecision | Unlikely | High |
| Febrile neutropenia | 37/1114 | 14/727 | 1.87 [1.03, 3.42] | Low | No inconsistency | No indirectness | No imprecision | Unlikely | High |
| Decreased appetite | 129/5034 | 38/3723 | 2.25 [1.57, 3.23] | Low | No inconsistency | No indirectness | No imprecision | Unlikely | High |
| Dyspnea | 54/2129 | 15/1207 | 1.91 [1.09, 3.35] | Low | No inconsistency | No indirectness | No imprecision | Unlikely | High |
| Hyponatraemia | 39/1554 | 19/1017 | 1.50 [0.86, 2.61] | Low | No inconsistency | No indirectness | No imprecision | Unlikely | High |
| Hypokalaemia | 38/1567 | 15/872 | 1.37 [0.76, 2.48] | Low | No inconsistency | No indirectness | No imprecision | Unlikely | High |
| Diarrhea | 95/4041 | 43/2975 | 1.46 [1.03, 2.08] | Low | No inconsistency | No indirectness | No imprecision | Unlikely | High |
| Lymphocyte count decreased | 27/1169 | 13/790 | 1.59 [0.84, 3.00] | Low | No inconsistency | No indirectness | No imprecision | Unlikely | High |
| Asthenia | 94/4327 | 64/3281 | 1.06 [0.77, 1.47] | Low | No inconsistency | No indirectness | No imprecision | Unlikely | High |
| Interstitial lung disease | 11/563 | 0/386 | 7.90 [0.99, 62.75] | Low | No inconsistency | No indirectness | No imprecision | Unlikely | High |
| Hepatic function abnormal | 10/525 | 3/366 | 2.18 [0.62, 7.62] | Low | No inconsistency | No indirectness | No imprecision | Unlikely | High |
| Hypomagnesaemia | 15/794 | 6/568 | 1.60 [0.63, 4.04] | Low | No inconsistency | No indirectness | No imprecision | Unlikely | High |
| Alanine aminotransferase increased | 62/3329 | 38/2211 | 1.10 [0.75, 1.61] | Low | No inconsistency | No indirectness | No imprecision | Unlikely | High |
| Hypertriglyceridaemia | 9/495 | 3/334 | 2.36 [0.69, 8.04] | Low | No inconsistency | No indirectness | No imprecision | Unlikely | High |
| Proteinuria | 15/871 | 12/691 | 1.24 [0.59, 2.57] | Low | No inconsistency | No indirectness | No imprecision | Unlikely | High |
| Gamma-glutamyltransferase increased | 23/1344 | 9/965 | 1.76 [0.83, 3.74] | Low | No inconsistency | No indirectness | No imprecision | Unlikely | High |
| Hyperglycemia | 22/1392 | 4/697 | 2.51 [0.92, 6.88] | Low | No inconsistency | No indirectness | No imprecision | Unlikely | High |
| Lymphopenia | 10/760 | 2/384 | 2.12 [0.54, 8.36] | Low | No inconsistency | No indirectness | No imprecision | Unlikely | High |
| Nausea | 66/5034 | 43/3723 | 1.07 [0.73, 1.56] | Low | No inconsistency | No indirectness | No imprecision | Unlikely | High |
| Vomiting | 59/4657 | 42/3345 | 0.98 [0.65, 1.47] | Low | No inconsistency | No indirectness | No imprecision | Unlikely | High |
| Arthralgia | 22/2210 | 3/1832 | 3.84 [1.43, 10.30] | Low | No inconsistency | No indirectness | No imprecision | Unlikely | High |
| Rash | 45/4545 | 14/3481 | 2.00 [1.19, 3.36] | Low | No inconsistency | No indirectness | No imprecision | Unlikely | High |
| Peripheral sensory neuropathy | 13/1384 | 6/1003 | 1.58 [0.61, 4.06] | Low | No inconsistency | No indirectness | No imprecision | Unlikely | High |
| Urinary tract infection | 7/771 | 1/387 | 2.52 [0.44, 14.46] | Low | No inconsistency | No indirectness | No imprecision | Unlikely | High |
| Hypertension | 7/771 | 2/387 | 1.51 [0.36, 6.29] | Low | No inconsistency | No indirectness | No imprecision | Unlikely | High |
| Aspartate aminotransferase increased | 30/3329 | 17/2211 | 1.13 [0.66, 1.95] | Low | No inconsistency | No indirectness | No imprecision | Unlikely | High |
| Pain in extremity | 22/2559 | 10/1531 | 1.20 [0.62, 2.34] | Low | No inconsistency | No indirectness | No imprecision | Unlikely | High |
| Hemoptysis | 12/1554 | 8/1017 | 1.10 [0.48, 2.55] | Low | No inconsistency | No indirectness | No imprecision | Unlikely | High |
| Renal failure | 5/771 | 0/387 | 3.02 [0.37, 24.99] | Low | No inconsistency | No indirectness | No imprecision | Unlikely | High |
| Blood alkaline phosphatase increased | 7/1083 | 0/541 | 4.02 [0.51, 32.04] | Low | No inconsistency | No indirectness | No imprecision | Unlikely | High |
| Blood creatinine increased | 7/1288 | 1/748 | 2.00 [0.47, 8.62] | Low | No inconsistency | No indirectness | No imprecision | Unlikely | High |
| Upper respiratory tract infection | 4/771 | 0/387 | 2.52 [0.30, 21.45] | Low | No inconsistency | No indirectness | No imprecision | Unlikely | High |
| Weight decreased | 8/1710 | 5/1043 | 0.87 [0.30, 2.47] | Low | No inconsistency | No indirectness | No imprecision | Unlikely | High |
| Edema peripheral | 7/1695 | 2/956 | 1.22 [0.35, 4.24] | Low | No inconsistency | No indirectness | No imprecision | Unlikely | High |
| Hypercholesteraemia | 2/495 | 0/334 | 2.14 [0.23, 19.82] | Low | No inconsistency | No indirectness | No imprecision | Unlikely | High |
| Cough | 8/2009 | 5/1319 | 1.01 [0.35, 2.90] | Low | No inconsistency | No indirectness | No imprecision | Unlikely | High |
| Stomatitis | 3/771 | 2/387 | 0.70 [0.14, 3.56] | Low | No inconsistency | No indirectness | No imprecision | Unlikely | High |
| Malaise | 3/798 | 4/459 | 0.42 [0.02, 7.17] | Low | Serious (-6) | No indirectness | No imprecision | Unlikely | Medium |
| Pyrexia | 9/2782 | 7/1892 | 0.91 [0.39, 2.15] | Low | No inconsistency | No indirectness | No imprecision | Unlikely | High |
| Blood bilirubin increased | 4/1405 | 2/871 | 1.01 [0.29, 3.53] | Low | No inconsistency | No indirectness | No imprecision | Unlikely | High |
| Pruritus | 4/1449 | 1/1239 | 1.91 [0.43, 8.51] | Low | No inconsistency | No indirectness | No imprecision | Unlikely | High |
| Hypoalbuminaemia | 6/2176 | 0/1235 | 2.72 [0.58, 12.88] | Low | No inconsistency | No indirectness | No imprecision | Unlikely | High |
| Constipation | 13/4841 | 10/3527 | 0.91 [0.41, 2.01] | Low | No inconsistency | No indirectness | No imprecision | Unlikely | High |
| Hypothyroidism | 6/2663 | 0/1802 | 1.96 [0.48, 7.95] | Low | No inconsistency | No indirectness | No imprecision | Unlikely | High |
| Hyperuricaemia | 1/484 | 1/331 | 1.00 [0.06, 15.86] | Low | No inconsistency | No indirectness | No imprecision | Unlikely | High |
| Hyperthyroidism | 1/760 | 1/384 | 0.51 [0.03, 8.05] | Low | No inconsistency | No indirectness | No imprecision | Unlikely | High |
| Abdominal pain upper | 1/771 | 1/387 | 0.50 [0.07, 3.57] | Low | No inconsistency | No indirectness | No imprecision | Unlikely | High |
| Headache | 1/789 | 2/565 | 0.48 [0.07, 3.38] | Low | No inconsistency | No indirectness | No imprecision | Unlikely | High |
| Myalgia | 1/794 | 0/568 | 1.52 [0.06, 37.16] | Low | No inconsistency | No indirectness | No imprecision | Unlikely | High |
| Alopecia | 4/3265 | 6/2548 | 0.60 [0.17, 2.10] | Low | No inconsistency | No indirectness | No imprecision | Unlikely | High |
| Hypoesthesia | 1/846 | 3/671 | 0.34 [0.05, 2.14] | Low | No inconsistency | No indirectness | No imprecision | Unlikely | High |
| Blood lactatedehydrogenase increased | 0/632 | 1/313 | 0.17 [0.01, 4.06] | Low | No inconsistency | No indirectness | No imprecision | Unlikely | High |
| **irAEs** |  |  |  |  |  |  |  |  |  |
| **Any grade** |  |  |  |  |  |  |  |  |  |
| Hypothyroidism | 529/4062 | 106/2888 | 5.84 [2.80, 12.17] | Low | Serious (-6) | No indirectness | No imprecision | Unlikely | Medium |
| Rash | 251/2580 | 139/1941 | 1.65 [0.89, 3.06] | Low | Serious (-6) | No indirectness | No imprecision | Unlikely | Medium |
| Hypokalemia | 29/429 | 16/277 | 1.71 [0.98, 3.00] | Low | No inconsistency | No indirectness | No imprecision | Unlikely | High |
| Pneumonitis | 238/3887 | 44/2713 | 3.63 [2.65, 4.97] | Low | No inconsistency | No indirectness | No imprecision | Unlikely | High |
| Pneumonia | 51/842 | 13/510 | 2.04 [1.15, 3.62] | Low | No inconsistency | No indirectness | No imprecision | Unlikely | High |
| Hepatitis | 154/3084 | 49/2225 | 2.28 [1.70, 3.08] | Low | No inconsistency | No indirectness | No imprecision | Unlikely | High |
| Hyperthyroidism | 188/4062 | 29/2888 | 4.06 [2.78, 5.92] | Low | No inconsistency | No indirectness | No imprecision | Unlikely | High |
| Aspartate aminotransferase increased | 43/933 | 13/466 | 1.65 [0.90, 3.03] | Low | No inconsistency | No indirectness | No imprecision | Unlikely | High |
| Alanine aminotransferase increased | 41/933 | 16/466 | 1.28 [0.73, 2.25] | Low | No inconsistency | No indirectness | No imprecision | Unlikely | High |
| Severe skin reactions | 97/2324 | 25/1841 | 2.63 [1.74, 3.97] | Low | No inconsistency | No indirectness | No imprecision | Unlikely | High |
| Amylase increased | 37/887 | 17/441 | 1.15 [0.34, 3.85] | Low | Serious (-6) | No indirectness | No imprecision | Unlikely | Medium |
| Pyrexia | 18/488 | 6/334 | 2.20 [0.87, 5.60] | Low | No inconsistency | No indirectness | No imprecision | Unlikely | High |
| Blood thyroid-stimulating hormone increased | 25/803 | 10/488 | 1.46 [0.69, 3.10] | Low | No inconsistency | No indirectness | No imprecision | Unlikely | High |
| Infusion reactions | 36/1323 | 13/1113 | 2.24 [1.21, 4.17] | Low | No inconsistency | No indirectness | No imprecision | Unlikely | High |
| Pruritus | 22/1112 | 11/644 | 1.24 [0.61, 2.50] | Low | No inconsistency | No indirectness | No imprecision | Unlikely | High |
| Diarrhea | 25/1450 | 12/981 | 1.23 [0.63, 2.41] | Low | No inconsistency | No indirectness | No imprecision | Unlikely | High |
| Colitis | 46/2775 | 5/2069 | 4.64 [2.18, 9.88] | Low | No inconsistency | No indirectness | No imprecision | Unlikely | High |
| Platelet count decreased | 8/537 | 6/357 | 0.94 [0.30, 2.98] | Low | No inconsistency | No indirectness | No imprecision | Unlikely | High |
| Diabetes | 29/2806 | 8/1944 | 1.88 [0.94, 3.78] | Low | No inconsistency | No indirectness | No imprecision | Unlikely | High |
| Meningoencephalitis | 10/1086 | 2/854 | 3.07 [0.85, 11.15] | Low | No inconsistency | No indirectness | No imprecision | Unlikely | High |
| Nephritis | 21/2324 | 3/1841 | 3.30 [1.21, 9.00] | Low | No inconsistency | No indirectness | No imprecision | Unlikely | High |
| Adrenal insufficiency | 19/2112 | 1/1678 | 4.58 [1.50, 13.98] | Low | No inconsistency | No indirectness | No imprecision | Unlikely | High |
| Proteinuria | 6/667 | 0/335 | 3.52 [0.44, 28.54] | Low | No inconsistency | No indirectness | No imprecision | Unlikely | High |
| Gamma-glutamyltransferase increased | 6/667 | 4/335 | 0.75 [0.21, 2.65] | Low | No inconsistency | No indirectness | No imprecision | Unlikely | High |
| Hypophysitis | 8/980 | 0/773 | 5.01 [0.91, 27.54] | Low | No inconsistency | No indirectness | No imprecision | Unlikely | High |
| Pancreatitis | 18/2463 | 3/1712 | 2.67 [1.05, 6.81] | Low | No inconsistency | No indirectness | No imprecision | Unlikely | High |
| Thyroiditis | 12/1689 | 1/1215 | 3.01 [0.98, 9.22] | Low | No inconsistency | No indirectness | No imprecision | Unlikely | High |
| Myocarditis | 12/2012 | 1/1376 | 2.43 [0.79, 7.49] | Low | No inconsistency | No indirectness | No imprecision | Unlikely | High |
| Vasculitis | 7/1323 | 0/1113 | 4.59 [0.84, 25.22] | Low | No inconsistency | No indirectness | No imprecision | Unlikely | High |
| Guillain-Barre syndrome | 1/1204 | 0/774 | 1.51 [0.06, 36.93] | Low | No inconsistency | No indirectness | No imprecision | Unlikely | High |
| **Grade 3-5** |  |  |  |  |  |  |  |  |  |
| Pneumonia | 58/842 | 1/510 | 4.45 [0.16, 125.23] | Low | Serious (-6) | No indirectness | No imprecision | Unlikely | Medium |
| Hepatitis | 69/3084 | 10/2225 | 3.77 [2.18, 6.53] | Low | No inconsistency | No indirectness | No imprecision | Unlikely | High |
| Pneumonitis | 68/3887 | 18/2713 | 2.40 [1.47, 3.94] | Low | No inconsistency | No indirectness | No imprecision | Unlikely | High |
| Rash | 26/2047 | 13/1587 | 1.55 [0.84, 2.88] | Low | No inconsistency | No indirectness | No imprecision | Unlikely | High |
| Severe skin reactions | 27/2324 | 5/1841 | 2.64 [1.22, 5.71] | Low | No inconsistency | No indirectness | No imprecision | Unlikely | High |
| Colitis | 27/2775 | 3/2069 | 3.84 [1.56, 9.42] | Low | No inconsistency | No indirectness | No imprecision | Unlikely | High |
| Hypothyroidism | 35/3887 | 5/2713 | 2.84 [1.31, 6.16] | Low | No inconsistency | No indirectness | No imprecision | Unlikely | High |
| Hypokalemia | 3/429 | 2/277 | 1.51 [0.26, 8.89] | Low | No inconsistency | No indirectness | No imprecision | Unlikely | High |
| Nephritis | 16/2324 | 2/1841 | 3.07 [1.08, 8.71] | Low | No inconsistency | No indirectness | No imprecision | Unlikely | High |
| Diabetes | 15/2806 | 3/1944 | 2.27 [0.85, 6.06] | Low | No inconsistency | No indirectness | No imprecision | Unlikely | High |
| Infusion reactions | 7/1323 | 2/1113 | 2.27 [0.60, 8.62] | Low | No inconsistency | No indirectness | No imprecision | Unlikely | High |
| Amylase increased | 4/887 | 1/441 | 1.48 [0.23, 9.36] | Low | No inconsistency | No indirectness | No imprecision | Unlikely | High |
| Pancreatitis | 8/2463 | 2/1712 | 1.73 [0.56, 5.29] | Low | No inconsistency | No indirectness | No imprecision | Unlikely | High |
| Alanine aminotransferase increased | 3/933 | 1/466 | 1.17 [0.17, 7.92] | Low | No inconsistency | No indirectness | No imprecision | Unlikely | High |
| Hypophysitis | 3/980 | 0/773 | 3.99 [0.45, 35.49] | Low | No inconsistency | No indirectness | No imprecision | Unlikely | High |
| Myocarditis | 6/2012 | 1/1376 | 1.78 [0.41, 7.76] | Low | No inconsistency | No indirectness | No imprecision | Unlikely | High |
| Meningoencephalitis | 3/1086 | 0/854 | 2.98 [0.36, 24.87] | Low | No inconsistency | No indirectness | No imprecision | Unlikely | High |
| Vasculitis | 3/1323 | 0/1113 | 2.40 [0.39, 14.84] | Low | No inconsistency | No indirectness | No imprecision | Unlikely | High |
| Platelet count decreased | 1/537 | 2/357 | 0.47 [0.07, 3.36] | Low | No inconsistency | No indirectness | No imprecision | Unlikely | High |
| Proteinuria | 1/667 | 0/335 | 1.52 [0.06, 37.08] | Low | No inconsistency | No indirectness | No imprecision | Unlikely | High |
| Gamma-glutamyltransferase increased | 1/667 | 0/335 | 1.52 [0.06, 37.08] | Low | No inconsistency | No indirectness | No imprecision | Unlikely | High |
| Adrenal insufficiency | 3/2112 | 1/1678 | 1.57 [0.31, 7.85] | Low | No inconsistency | No indirectness | No imprecision | Unlikely | High |
| Diarrhea | 2/1450 | 1/981 | 0.90 [0.19, 4.19] | Low | No inconsistency | No indirectness | No imprecision | Unlikely | High |
| Hyperthyroidism | 5/3887 | 0/2713 | 2.62 [0.63, 10.81] | Low | No inconsistency | No indirectness | No imprecision | Unlikely | High |
| Thyroiditis | 2/1689 | 0/1215 | 3.03 [0.32, 28.97] | Low | No inconsistency | No indirectness | No imprecision | Unlikely | High |
| Aspartate aminotransferase increased | 1/933 | 0/466 | 1.50 [0.06, 36.74] | Low | No inconsistency | No indirectness | No imprecision | Unlikely | High |
| Guillain-Barre syndrome | 1/1204 | 0/774 | 1.51 [0.06, 36.93] | Low | No inconsistency | No indirectness | No imprecision | Unlikely | High |

**Abbreviations:** AE: Adverse event; ALT: Alanine aminotransferase; AST: Aspartate aminotransferase; CI: Confidence interval; CPS: Combined positive score; CR: Complete response; DCR: Disease control rate; DOR: Duration of response; DORR: Duration of response rate; ECOG PS: Eastern Cooperative Oncology Group Performance Status; GRADE: Grading of Recommendations Assessment, Development, and Evaluation; HR: Hazard ratio; irAE: Immune-related adverse event; NSCLC: Non-small-cell lung cancer; ORR: Objective response rate; OS: Overall survival; OSR: Overall survival rate; PC: PD-1/PD-L1 inhibitors combined with chemotherapy; PD: Progressive disease; PD-1: Programmed cell death protein 1; PD-L1: Programmed death-ligand 1; PFS: Progression-free survival; PFSR: Progression-free survival rate; PR: Partial response; RCT: Randomized controlled trial; RR: Risk ratio; SD: Stable disease; TEAE: Treatment-emergent adverse event; TRAE: Treatment-related adverse event.

^a^ Differences: HR for OS, PFS and DOR; RR for OSR, PFSR, DORR, responses, and AEs.

^b^ Risk of bias assessed using the Jadad scale for randomized controlled trials.

^c^ Publication bias was explored through visual inspection of the funnel plots.
