# Supplementary material for: The benefits and risks of adding PD-1/PD-L1 inhibitors to chemotherapy for stage IIIb-IV non-small-cell lung cancer: an updated meta-analysis based on phase 3 randomized controlled trials
Source: Front Oncol. 2025 Sep 11;15:1590017. doi: 10.3389/fonc.2025.1590017 (PMC12460147; doi:10.3389/fonc.2025.1590017)
Supplement: Supplementary file 12 [file Table4.doc]

**Table S4** Any grade treatment-emergent adverse events.

| **TEAEs** | **PC** | |  | **Chemotherapy** | | **Risk ratio [95% CI]** | **P** |
| --- | --- | --- | --- | --- | --- | --- | --- |
|  | **Event/total** | **%** |  | **Event/total** | **%** |  |  |
| Anemia | 2933/5034 | 58.26% |  | 2076/3723 | 55.76% | 1.03 [0.99, 1.06] | 0.14 |
| Neutrophil count decreased | 1372/3502 | 39.18% |  | 1067/2815 | 37.90% | 1.04 [0.98, 1.09] | 0.19 |
| White blood cell decreased | 1215/3299 | 36.83% |  | 914/2446 | 37.37% | 1.05 [0.99, 1.11] | 0.08 |
| Nausea | 1725/5034 | 34.27% |  | 1169/3723 | 31.40% | 1.07 [1.00, 1.13] | 0.03 |
| Neutropenia | 1340/4016 | 33.37% |  | 856/2836 | 30.18% | 1.05 [0.98, 1.12] | 0.14 |
| Alopecia | 1001/3265 | 30.66% |  | 754/2548 | 29.59% | 1.09 [1.02, 1.17] | 0.02 |
| Platelet count decreased | 893/3099 | 28.82% |  | 628/2254 | 27.86% | 1.10 [1.01, 1.19] | 0.03 |
| Fatigue | 1055/4071 | 25.92% |  | 632/2890 | 21.87% | 1.12 [1.03, 1.22] | 0.009 |
| Alanine aminotransferase increased | 857/3329 | 25.74% |  | 515/2211 | 23.29% | 1.11 [1.01, 1.22] | 0.03 |
| Leukopenia | 711/2774 | 25.63% |  | 401/1785 | 22.46% | 1.06 [0.97, 1.15] | 0.19 |
| Thrombocytopenia | 970/3839 | 25.27% |  | 588/2650 | 22.19% | 1.07 [0.99, 1.17] | 0.09 |
| Aspartate aminotransferase increased | 811/3329 | 24.36% |  | 429/2211 | 19.40% | 1.25 [1.13, 1.38] | < 0.00001 |
| Decreased appetite | 1208/5034 | 24.00% |  | 828/3723 | 22.24% | 1.09 [1.01, 1.18] | 0.03 |
| Hypoesthesia | 183/846 | 21.63% |  | 146/671 | 21.76% | 1.19 [0.99, 1.43] | 0.06 |
| Constipation | 1039/4841 | 21.46% |  | 673/3527 | 19.08% | 1.09 [1.00, 1.19] | 0.04 |
| Cough | 419/2009 | 20.86% |  | 220/1319 | 16.68% | 1.19 [0.90, 1.58] | 0.22 |
| Diarrhea | 777/4041 | 19.23% |  | 428/2975 | 14.39% | 1.30 [1.17, 1.45] | < 0.00001 |
| Vomiting | 876/4657 | 18.81% |  | 543/3345 | 16.23% | 1.13 [1.02, 1.24] | 0.02 |
| Asthenia | 809/4327 | 18.70% |  | 587/3281 | 17.89% | 1.05 [0.96, 1.16] | 0.29 |
| Pyrexia | 467/2782 | 16.79% |  | 237/1892 | 12.53% | 1.31 [1.14, 1.52] | 0.0002 |
| Hypomagnesaemia | 133/794 | 16.75% |  | 65/568 | 11.44% | 1.30 [0.99, 1.72] | 0.06 |
| Pain in extremity | 406/2559 | 15.87% |  | 211/1531 | 13.78% | 1.27 [0.98, 1.65] | 0.07 |
| Dyspnea | 333/2129 | 15.64% |  | 166/1207 | 13.75% | 1.13 [0.82, 1.55] | 0.45 |
| Hypoalbuminaemia | 330/2176 | 15.17% |  | 142/1235 | 11.50% | 1.38 [1.15, 1.66] | 0.0005 |
| Rash | 676/4545 | 14.87% |  | 244/3481 | 7.01% | 2.07 [1.81, 2.38] | < 0.00001 |
| Arthralgia | 315/2210 | 14.25% |  | 195/1832 | 10.64% | 1.31 [1.11, 1.55] | 0.001 |
| Edema peripheral | 241/1695 | 14.22% |  | 98/956 | 10.25% | 1.36 [1.09, 1.69] | 0.006 |
| Peripheral sensory neuropathy | 195/1384 | 14.09% |  | 116/1003 | 11.57% | 1.29 [1.04, 1.60] | 0.02 |
| Weight decreased | 222/1710 | 12.98% |  | 143/1043 | 13.71% | 0.95 [0.70, 1.30] | 0.77 |
| Pruritus | 185/1449 | 12.77% |  | 57/1239 | 4.60% | 3.07 [1.76, 5.36] | < 0.0001 |
| Headache | 100/789 | 12.67% |  | 48/565 | 8.50% | 1.27 [0.70, 2.32] | 0.43 |
| Hypokalaemia | 186/1567 | 11.87% |  | 84/872 | 9.63% | 1.26 [0.99, 1.61] | 0.06 |
| Hyperuricaemia | 57/484 | 11.78% |  | 36/331 | 10.88% | 1.11 [0.54, 2.28] | 0.77 |
| Hepatic function abnormal | 59/525 | 11.24% |  | 36/366 | 9.84% | 1.45 [0.98, 2.14] | 0.06 |
| Hypertriglyceridaemia | 55/495 | 11.11% |  | 33/334 | 9.88% | 1.50 [1.02, 2.23] | 0.04 |
| Hyperglycemia | 151/1392 | 10.85% |  | 52/697 | 7.46% | 1.45 [1.08, 1.95] | 0.01 |
| Hyponatraemia | 165/1554 | 10.62% |  | 103/1017 | 10.13% | 1.23 [0.97, 1.56] | 0.09 |
| Hypothyroidism | 274/2663 | 10.29% |  | 27/1802 | 1.50% | 6.39 [4.33, 9.42] | < 0.00001 |
| Gamma-glutamyltransferase increased | 134/1344 | 9.97% |  | 89/965 | 9.22% | 1.22 [0.81, 1.85] | 0.34 |
| Pneumonia | 205/2135 | 9.60% |  | 114/1424 | 8.01% | 1.25 [1.01, 1.55] | 0.04 |
| Blood creatinine increased | 120/1288 | 9.32% |  | 36/748 | 4.81% | 2.09 [1.46, 3.01] | < 0.0001 |
| Hemoptysis | 140/1554 | 9.01% |  | 84/1017 | 8.26% | 1.24 [0.96, 1.61] | 0.10 |
| Urinary tract infection | 67/771 | 8.69% |  | 22/387 | 5.68% | 1.54 [0.97, 2.43] | 0.07 |
| Proteinuria | 73/871 | 8.38% |  | 49/691 | 7.09% | 1.43 [0.71, 2.87] | 0.32 |
| Blood bilirubin increased | 111/1405 | 7.90% |  | 58/871 | 6.66% | 1.33 [0.83, 2.12] | 0.23 |
| Hyperthyroidism | 60/760 | 7.89% |  | 4/384 | 1.04% | 7.58 [2.78, 20.64] | < 0.0001 |
| Malaise | 60/798 | 7.52% |  | 36/459 | 7.84% | 1.15 [0.78, 1.71] | 0.47 |
| Myalgia | 56/794 | 7.05% |  | 25/568 | 4.40% | 1.49 [0.92, 2.40] | 0.11 |
| Lymphocyte count decreased | 77/1169 | 6.59% |  | 45/790 | 5.70% | 1.39 [0.97, 1.99] | 0.07 |
| Hypercholesteraemia | 32/495 | 6.46% |  | 15/334 | 4.49% | 1.87 [1.03, 3.39] | 0.04 |
| Stomatitis | 43/771 | 5.58% |  | 12/387 | 3.10% | 1.76 [0.95, 3.24] | 0.07 |
| Blood lactatedehydrogenase increased | 35/632 | 5.54% |  | 9/313 | 2.88% | 1.93 [0.94, 3.95] | 0.07 |
| Myelosuppression | 29/525 | 5.52% |  | 14/366 | 3.83% | 1.37 [0.37, 5.12] | 0.64 |
| Upper respiratory tract infection | 40/771 | 5.19% |  | 12/387 | 3.10% | 1.64 [0.88, 3.03] | 0.12 |
| Blood alkaline phosphatase increased | 56/1083 | 5.17% |  | 24/541 | 4.44% | 1.16 [0.73, 1.86] | 0.52 |
| Lymphopenia | 39/760 | 5.13% |  | 23/384 | 5.99% | 0.86 [0.53, 1.39] | 0.53 |
| Abdominal pain upper | 33/771 | 4.28% |  | 12/387 | 3.10% | 1.89 [0.32, 11.30] | 0.49 |
| Hypertension | 28/771 | 3.63% |  | 10/387 | 2.58% | 1.41 [0.69, 2.87] | 0.34 |
| Febrile neutropenia | 37/1114 | 3.32% |  | 15/727 | 2.06% | 1.73 [0.97, 3.10] | 0.06 |
| Interstitial lung disease | 18/563 | 3.20% |  | 1/386 | 0.26% | 7.75 [1.50, 40.19] | 0.01 |
| Renal failure | 13/771 | 1.69% |  | 4/387 | 1.03% | 1.51 [0.52, 4.36] | 0.44 |

**Abbreviations:** AE: Adverse event; ALT: Alanine aminotransferase; AST: Aspartate aminotransferase; CI: Confidence interval; PC: PD-1/PD-L1 inhibitors combined with chemotherapy; PD-1: Programmed cell death protein 1; PD-L1: Programmed death-ligand 1; RR: Risk ratio; TEAE: Treatment-emergent adverse event.
