# Supplementary material for: The benefits and risks of adding PD-1/PD-L1 inhibitors to chemotherapy for stage IIIb-IV non-small-cell lung cancer: an updated meta-analysis based on phase 3 randomized controlled trials
Source: Front Oncol. 2025 Sep 11;15:1590017. doi: 10.3389/fonc.2025.1590017 (PMC12460147; doi:10.3389/fonc.2025.1590017)
Supplement: Supplementary file 13 [file Table5.doc]

**Table S5** Grade 3-5 treatment-emergent adverse events.

| **Grade 3-5 TEAEs** | **PC** | |  | **Chemotherapy** | | **Risk ratio [95% CI]** | **P** |
| --- | --- | --- | --- | --- | --- | --- | --- |
|  | **Event/total** | **%** |  | **Event/total** | **%** |  |  |
| Neutrophil count decreased | 834/3502 | 23.81% |  | 652/2815 | 23.16% | 1.05 [0.96, 1.14] | 0.27 |
| Neutropenia | 814/4016 | 20.27% |  | 515/2836 | 18.16% | 1.07 [0.97, 1.18] | 0.16 |
| Anemia | 895/5034 | 17.78% |  | 622/3723 | 16.71% | 1.04 [0.95, 1.14] | 0.42 |
| White blood cell decreased | 418/3299 | 12.67% |  | 310/2446 | 12.67% | 1.09 [0.95, 1.24] | 0.22 |
| Platelet count decreased | 296/3099 | 9.55% |  | 195/2254 | 8.65% | 1.19 [1.01, 1.40] | 0.04 |
| Leukopenia | 250/2774 | 9.01% |  | 146/1785 | 8.18% | 1.02 [0.85, 1.23] | 0.79 |
| Thrombocytopenia | 291/3839 | 7.58% |  | 165/2650 | 6.23% | 1.17 [0.98, 1.41] | 0.09 |
| Pneumonia | 102/2135 | 4.78% |  | 61/1424 | 4.28% | 1.15 [0.85, 1.56] | 0.37 |
| Fatigue | 148/4071 | 3.64% |  | 69/2890 | 2.39% | 1.49 [1.13, 1.98] | 0.005 |
| Myelosuppression | 19/525 | 3.62% |  | 7/366 | 1.91% | 2.18 [0.96, 4.95] | 0.06 |
| Febrile neutropenia | 37/1114 | 3.32% |  | 14/727 | 1.93% | 1.87 [1.03, 3.42] | 0.04 |
| Decreased appetite | 129/5034 | 2.56% |  | 38/3723 | 1.02% | 2.25 [1.57, 3.23] | < 0.00001 |
| Dyspnea | 54/2129 | 2.54% |  | 15/1207 | 1.24% | 1.91 [1.09, 3.35] | 0.02 |
| Hyponatraemia | 39/1554 | 2.51% |  | 19/1017 | 1.87% | 1.50 [0.86, 2.61] | 0.16 |
| Hypokalaemia | 38/1567 | 2.43% |  | 15/872 | 1.72% | 1.37 [0.76, 2.48] | 0.30 |
| Diarrhea | 95/4041 | 2.35% |  | 43/2975 | 1.45% | 1.46 [1.03, 2.08] | 0.03 |
| Lymphocyte count decreased | 27/1169 | 2.31% |  | 13/790 | 1.65% | 1.59 [0.84, 3.00] | 0.15 |
| Asthenia | 94/4327 | 2.17% |  | 64/3281 | 1.95% | 1.06 [0.77, 1.47] | 0.70 |
| Interstitial lung disease | 11/563 | 1.95% |  | 0/386 | 0.00% | 7.90 [0.99, 62.75] | 0.05 |
| Hepatic function abnormal | 10/525 | 1.90% |  | 3/366 | 0.82% | 2.18 [0.62, 7.62] | 0.22 |
| Hypomagnesaemia | 15/794 | 1.89% |  | 6/568 | 1.06% | 1.60 [0.63, 4.04] | 0.32 |
| Alanine aminotransferase increased | 62/3329 | 1.86% |  | 38/2211 | 1.72% | 1.10 [0.75, 1.61] | 0.63 |
| Hypertriglyceridaemia | 9/495 | 1.82% |  | 3/334 | 0.90% | 2.36 [0.69, 8.04] | 0.17 |
| Proteinuria | 15/871 | 1.72% |  | 12/691 | 1.74% | 1.24 [0.59, 2.57] | 0.57 |
| Gamma-glutamyltransferase increased | 23/1344 | 1.71% |  | 9/965 | 0.93% | 1.76 [0.83, 3.74] | 0.14 |
| Hyperglycemia | 22/1392 | 1.58% |  | 4/697 | 0.57% | 2.51 [0.92, 6.88] | 0.07 |
| Lymphopenia | 10/760 | 1.32% |  | 2/384 | 0.52% | 2.12 [0.54, 8.36] | 0.28 |
| Nausea | 66/5034 | 1.31% |  | 43/3723 | 1.15% | 1.07 [0.73, 1.56] | 0.72 |
| Vomiting | 59/4657 | 1.27% |  | 42/3345 | 1.26% | 0.98 [0.65, 1.47] | 0.92 |
| Arthralgia | 22/2210 | 1.00% |  | 3/1832 | 0.16% | 3.84 [1.43, 10.30] | 0.008 |
| Rash | 45/4545 | 0.99% |  | 14/3481 | 0.40% | 2.00 [1.19, 3.36] | 0.009 |
| Peripheral sensory neuropathy | 13/1384 | 0.94% |  | 6/1003 | 0.60% | 1.58 [0.61, 4.06] | 0.34 |
| Urinary tract infection | 7/771 | 0.91% |  | 1/387 | 0.26% | 2.52 [0.44, 14.46] | 0.30 |
| Hypertension | 7/771 | 0.91% |  | 2/387 | 0.52% | 1.51 [0.36, 6.29] | 0.57 |
| Aspartate aminotransferase increased | 30/3329 | 0.90% |  | 17/2211 | 0.77% | 1.13 [0.66, 1.95] | 0.65 |
| Pain in extremity | 22/2559 | 0.86% |  | 10/1531 | 0.65% | 1.20 [0.62, 2.34] | 0.59 |
| Hemoptysis | 12/1554 | 0.77% |  | 8/1017 | 0.79% | 1.10 [0.48, 2.55] | 0.82 |
| Renal failure | 5/771 | 0.65% |  | 0/387 | 0.00% | 3.02 [0.37, 24.99] | 0.30 |
| Blood alkaline phosphatase increased | 7/1083 | 0.65% |  | 0/541 | 0.00% | 4.02 [0.51, 32.04] | 0.19 |
| Blood creatinine increased | 7/1288 | 0.54% |  | 1/748 | 0.13% | 2.00 [0.47, 8.62] | 0.35 |
| Upper respiratory tract infection | 4/771 | 0.52% |  | 0/387 | 0.00% | 2.52 [0.30, 21.45] | 0.40 |
| Weight decreased | 8/1710 | 0.47% |  | 5/1043 | 0.48% | 0.87 [0.30, 2.47] | 0.79 |
| Edema peripheral | 7/1695 | 0.41% |  | 2/956 | 0.21% | 1.22 [0.35, 4.24] | 0.75 |
| Hypercholesteraemia | 2/495 | 0.40% |  | 0/334 | 0.00% | 2.14 [0.23, 19.82] | 0.50 |
| Cough | 8/2009 | 0.40% |  | 5/1319 | 0.38% | 1.01 [0.35, 2.90] | 0.99 |
| Stomatitis | 3/771 | 0.39% |  | 2/387 | 0.52% | 0.70 [0.14, 3.56] | 0.67 |
| Malaise | 3/798 | 0.38% |  | 4/459 | 0.87% | 0.42 [0.02, 7.17] | 0.55 |
| Pyrexia | 9/2782 | 0.32% |  | 7/1892 | 0.37% | 0.91 [0.39, 2.15] | 0.83 |
| Blood bilirubin increased | 4/1405 | 0.28% |  | 2/871 | 0.23% | 1.01 [0.29, 3.53] | 0.99 |
| Pruritus | 4/1449 | 0.28% |  | 1/1239 | 0.08% | 1.91 [0.43, 8.51] | 0.39 |
| Hypoalbuminaemia | 6/2176 | 0.28% |  | 0/1235 | 0.00% | 2.72 [0.58, 12.88] | 0.21 |
| Constipation | 13/4841 | 0.27% |  | 10/3527 | 0.28% | 0.91 [0.41, 2.01] | 0.82 |
| Hypothyroidism | 6/2663 | 0.23% |  | 0/1802 | 0.00% | 1.96 [0.48, 7.95] | 0.35 |
| Hyperuricaemia | 1/484 | 0.21% |  | 1/331 | 0.30% | 1.00 [0.06, 15.86] | 1.00 |
| Hyperthyroidism | 1/760 | 0.13% |  | 1/384 | 0.26% | 0.51 [0.03, 8.05] | 0.63 |
| Abdominal pain upper | 1/771 | 0.13% |  | 1/387 | 0.26% | 0.50 [0.07, 3.57] | 0.49 |
| Headache | 1/789 | 0.13% |  | 2/565 | 0.35% | 0.48 [0.07, 3.38] | 0.46 |
| Myalgia | 1/794 | 0.13% |  | 0/568 | 0.00% | 1.52 [0.06, 37.16] | 0.80 |
| Alopecia | 4/3265 | 0.12% |  | 6/2548 | 0.24% | 0.60 [0.17, 2.10] | 0.43 |
| Hypoesthesia | 1/846 | 0.12% |  | 3/671 | 0.45% | 0.34 [0.05, 2.14] | 0.25 |
| Blood lactatedehydrogenase increased | 0/632 | 0.00% |  | 1/313 | 0.32% | 0.17 [0.01, 4.06] | 0.27 |

**Abbreviations:** AE: Adverse event; ALT: Alanine aminotransferase; AST: Aspartate aminotransferase; CI: Confidence interval; PC: PD-1/PD-L1 inhibitors combined with chemotherapy; PD-1: Programmed cell death protein 1; PD-L1: Programmed death-ligand 1; RR: Risk ratio; TEAE: Treatment-emergent adverse event.
