# Supplementary material for: The benefits and risks of adding PD-1/PD-L1 inhibitors to chemotherapy for stage IIIb-IV non-small-cell lung cancer: an updated meta-analysis based on phase 3 randomized controlled trials
Source: Front Oncol. 2025 Sep 11;15:1590017. doi: 10.3389/fonc.2025.1590017 (PMC12460147; doi:10.3389/fonc.2025.1590017)
Supplement: Supplementary file 14 [file Table6.doc]

**Table S6** Any grade immune-related adverse events.

| **irAEs** | **PC** | |  | **Chemotherapy** | | **Risk ratio [95% CI]** | **P** |
| --- | --- | --- | --- | --- | --- | --- | --- |
|  | **Event/total** | **%** |  | **Event/total** | **%** |  |  |
| Hypothyroidism | 529/4062 | 13.02% |  | 106/2888 | 3.67% | 5.84 [2.80, 12.17] | < 0.00001 |
| Rash | 251/2580 | 9.73% |  | 139/1941 | 7.16% | 1.65 [0.89, 3.06] | 0.11 |
| Hypokalemia | 29/429 | 6.76% |  | 16/277 | 5.78% | 1.71 [0.98, 3.00] | 0.06 |
| Pneumonitis | 238/3887 | 6.12% |  | 44/2713 | 1.62% | 3.63 [2.65, 4.97] | < 0.00001 |
| Pneumonia | 51/842 | 6.06% |  | 13/510 | 2.55% | 2.04 [1.15, 3.62] | 0.01 |
| Hepatitis | 154/3084 | 4.99% |  | 49/2225 | 2.20% | 2.28 [1.70, 3.08] | < 0.00001 |
| Hyperthyroidism | 188/4062 | 4.63% |  | 29/2888 | 1.00% | 4.06 [2.78, 5.92] | < 0.00001 |
| Aspartate aminotransferase increased | 43/933 | 4.61% |  | 13/466 | 2.79% | 1.65 [0.90, 3.03] | 0.11 |
| Alanine aminotransferase increased | 41/933 | 4.39% |  | 16/466 | 3.43% | 1.28 [0.73, 2.25] | 0.39 |
| Severe skin reactions | 97/2324 | 4.17% |  | 25/1841 | 1.36% | 2.63 [1.74, 3.97] | < 0.00001 |
| Amylase increased | 37/887 | 4.17% |  | 17/441 | 3.85% | 1.15 [0.34, 3.85] | 0.82 |
| Pyrexia | 18/488 | 3.69% |  | 6/334 | 1.80% | 2.20 [0.87, 5.60] | 0.10 |
| Blood thyroid-stimulating hormone increased | 25/803 | 3.11% |  | 10/488 | 2.05% | 1.46 [0.69, 3.10] | 0.32 |
| Infusion reactions | 36/1323 | 2.72% |  | 13/1113 | 1.17% | 2.24 [1.21, 4.17] | 0.01 |
| Pruritus | 22/1112 | 1.98% |  | 11/644 | 1.71% | 1.24 [0.61, 2.50] | 0.55 |
| Diarrhea | 25/1450 | 1.72% |  | 12/981 | 1.22% | 1.23 [0.63, 2.41] | 0.54 |
| Colitis | 46/2775 | 1.66% |  | 5/2069 | 0.24% | 4.64 [2.18, 9.88] | < 0.0001 |
| Platelet count decreased | 8/537 | 1.49% |  | 6/357 | 1.68% | 0.94 [0.30, 2.98] | 0.92 |
| Diabetes | 29/2806 | 1.03% |  | 8/1944 | 0.41% | 1.88 [0.94, 3.78] | 0.08 |
| Meningoencephalitis | 10/1086 | 0.92% |  | 2/854 | 0.23% | 3.07 [0.85, 11.15] | 0.09 |
| Nephritis | 21/2324 | 0.90% |  | 3/1841 | 0.16% | 3.30 [1.21, 9.00] | 0.02 |
| Adrenal insufficiency | 19/2112 | 0.90% |  | 1/1678 | 0.06% | 4.58 [1.50, 13.98] | 0.007 |
| Proteinuria | 6/667 | 0.90% |  | 0/335 | 0.00% | 3.52 [0.44, 28.54] | 0.24 |
| Gamma-glutamyltransferase increased | 6/667 | 0.90% |  | 4/335 | 1.19% | 0.75 [0.21, 2.65] | 0.66 |
| Hypophysitis | 8/980 | 0.82% |  | 0/773 | 0.00% | 5.01 [0.91, 27.54] | 0.06 |
| Pancreatitis | 18/2463 | 0.73% |  | 3/1712 | 0.18% | 2.67 [1.05, 6.81] | 0.04 |
| Thyroiditis | 12/1689 | 0.71% |  | 1/1215 | 0.08% | 3.01 [0.98, 9.22] | 0.05 |
| Myocarditis | 12/2012 | 0.60% |  | 1/1376 | 0.07% | 2.43 [0.79, 7.49] | 0.12 |
| Vasculitis | 7/1323 | 0.53% |  | 0/1113 | 0.00% | 4.59 [0.84, 25.22] | 0.08 |
| Guillain-Barre syndrome | 1/1204 | 0.08% |  | 0/774 | 0.00% | 1.51 [0.06, 36.93] | 0.80 |

**Abbreviations:** AE: Adverse event; ALT: Alanine aminotransferase; AST: Aspartate aminotransferase; CI: Confidence interval; irAE: Immune-related adverse event; PC: PD-1/PD-L1 inhibitors combined with chemotherapy; PD-1: Programmed cell death protein 1; PD-L1: Programmed death-ligand 1; RR: Risk ratio.
