# Supplementary material for: The benefits and risks of adding PD-1/PD-L1 inhibitors to chemotherapy for stage IIIb-IV non-small-cell lung cancer: an updated meta-analysis based on phase 3 randomized controlled trials
Source: Front Oncol. 2025 Sep 11;15:1590017. doi: 10.3389/fonc.2025.1590017 (PMC12460147; doi:10.3389/fonc.2025.1590017)
Supplement: Supplementary file 15 [file Table7.doc]

**Table S7** Grade 3-5 immune-related adverse events.

| **Grade 3-5 irAEs** | **PC** | |  | **Chemotherapy** | | **Risk ratio [95% CI]** | **P** |
| --- | --- | --- | --- | --- | --- | --- | --- |
|  | **Event/total** | **%** |  | **Event/total** | **%** |  |  |
| Pneumonia | 58/842 | 6.89% |  | 1/510 | 0.20% | 4.45 [0.16, 125.23] | 0.38 |
| Hepatitis | 69/3084 | 2.24% |  | 10/2225 | 0.45% | 3.77 [2.18, 6.53] | < 0.00001 |
| Pneumonitis | 68/3887 | 1.75% |  | 18/2713 | 0.66% | 2.40 [1.47, 3.94] | 0.0005 |
| Rash | 26/2047 | 1.27% |  | 13/1587 | 0.82% | 1.55 [0.84, 2.88] | 0.16 |
| Severe skin reactions | 27/2324 | 1.16% |  | 5/1841 | 0.27% | 2.64 [1.22, 5.71] | 0.01 |
| Colitis | 27/2775 | 0.97% |  | 3/2069 | 0.14% | 3.84 [1.56, 9.42] | 0.003 |
| Hypothyroidism | 35/3887 | 0.90% |  | 5/2713 | 0.18% | 2.84 [1.31, 6.16] | 0.008 |
| Hypokalemia | 3/429 | 0.70% |  | 2/277 | 0.72% | 1.51 [0.26, 8.89] | 0.65 |
| Nephritis | 16/2324 | 0.69% |  | 2/1841 | 0.11% | 3.07 [1.08, 8.71] | 0.04 |
| Diabetes | 15/2806 | 0.53% |  | 3/1944 | 0.15% | 2.27 [0.85, 6.06] | 0.10 |
| Infusion reactions | 7/1323 | 0.53% |  | 2/1113 | 0.18% | 2.27 [0.60, 8.62] | 0.23 |
| Amylase increased | 4/887 | 0.45% |  | 1/441 | 0.23% | 1.48 [0.23, 9.36] | 0.68 |
| Pancreatitis | 8/2463 | 0.32% |  | 2/1712 | 0.12% | 1.73 [0.56, 5.29] | 0.34 |
| Alanine aminotransferase increased | 3/933 | 0.32% |  | 1/466 | 0.21% | 1.17 [0.17, 7.92] | 0.87 |
| Hypophysitis | 3/980 | 0.31% |  | 0/773 | 0.00% | 3.99 [0.45, 35.49] | 0.21 |
| Myocarditis | 6/2012 | 0.30% |  | 1/1376 | 0.07% | 1.78 [0.41, 7.76] | 0.45 |
| Meningoencephalitis | 3/1086 | 0.28% |  | 0/854 | 0.00% | 2.98 [0.36, 24.87] | 0.31 |
| Vasculitis | 3/1323 | 0.23% |  | 0/1113 | 0.00% | 2.40 [0.39, 14.84] | 0.35 |
| Platelet count decreased | 1/537 | 0.19% |  | 2/357 | 0.56% | 0.47 [0.07, 3.36] | 0.45 |
| Proteinuria | 1/667 | 0.15% |  | 0/335 | 0.00% | 1.52 [0.06, 37.08] | 0.80 |
| Gamma-glutamyltransferase increased | 1/667 | 0.15% |  | 0/335 | 0.00% | 1.52 [0.06, 37.08] | 0.80 |
| Adrenal insufficiency | 3/2112 | 0.14% |  | 1/1678 | 0.06% | 1.57 [0.31, 7.85] | 0.58 |
| Diarrhea | 2/1450 | 0.14% |  | 1/981 | 0.10% | 0.90 [0.19, 4.19] | 0.89 |
| Hyperthyroidism | 5/3887 | 0.13% |  | 0/2713 | 0.00% | 2.62 [0.63, 10.81] | 0.18 |
| Thyroiditis | 2/1689 | 0.12% |  | 0/1215 | 0.00% | 3.03 [0.32, 28.97] | 0.34 |
| Aspartate aminotransferase increased | 1/933 | 0.11% |  | 0/466 | 0.00% | 1.50 [0.06, 36.74] | 0.80 |
| Guillain-Barre syndrome | 1/1204 | 0.08% |  | 0/774 | 0.00% | 1.51 [0.06, 36.93] | 0.80 |

**Abbreviations:** AE: Adverse event; ALT: Alanine aminotransferase; AST: Aspartate aminotransferase; CI: Confidence interval; irAE: Immune-related adverse event; PC: PD-1/PD-L1 inhibitors combined with chemotherapy; PD-1: Programmed cell death protein 1; PD-L1: Programmed death-ligand 1; RR: Risk ratio.
